# Supplementary material for: Multi-omics analyses reveal that the gut microbiome and its metabolites promote milk fat synthesis in Zhongdian yak cows
Source: PeerJ. 2022 Dec 2;10:e14444. doi: 10.7717/peerj.14444 (PMC9744170; doi:10.7717/peerj.14444)
Supplement: Supplemental Information 13 [file peerj-10-14444-s013.zip › Web_Report/HTML/meta_lipidmaps_anno.xls.html]

meta\_lipidmaps\_anno.xls


## meta\_lipidmaps\_anno.xls

| #ID | Name | LM\_ID | NAME | SYSTEMATIC\_NAME | SYNONYMS | CATEGORY | MAIN\_CLASS | SUB\_CLASS | ABBREVIATION |
| --- | --- | --- | --- | --- | --- | --- | --- | --- | --- |
| pos\_10 | Deoxycholic acid | LMST04010040 | Deoxycholic acid | 3alpha,12alpha-Dihydroxy-5beta-cholan-24-oic Acid | - | Sterol Lipids [ST] | Bile acids and derivatives [ST04] | C24 bile acids, alcohols, and derivatives [ST0401] | ST 24:1;O4 |
| pos\_100 | Dihydroscoparin | LMPK12140433 | Dihydroscoparin | - | - | Polyketides [PK] | Flavonoids [PK12] | Flavanones [PK1214] | - |
| pos\_1003 | Testolactone | LMST02020084 | testolactone | 3-oxo-13,17-secoandrosta-1,4-dieno-17,13alpha-lactone | (4aS,4bR,10aR,10bS,12aS)-10a,12a-dimethyl-3,4,4a,5,6,10a,10b,11,12,12a-decahydro-2H-naphtho[2,1-f]chromene-2,8(4bH)-dione; 1,2-didehydrotestololactone; 1-dehydrotestololactone; 13-hydroxy-3-oxo-13,17-secoandrosta-1,4-dien-17-oic acid delta-lactone; D-homo-17a-oxaandrosta-1,4-diene-3,17-dione; Delta(1)-testololactone; Teslac; Testolactone | Sterol Lipids [ST] | Steroids [ST02] | C19 steroids (androgens) and derivatives [ST0202] | ST 19:4;O3 |
| pos\_1005 | Bovinic acid | LMFA01030118 | Rumenic acid | 9Z,11E-octadecadienoic acid | cis-9, trans-11-octadecadienoic acid; bovinic acid; C18:2n-7,9 | Fatty Acyls [FA] | Fatty Acids and Conjugates [FA01] | Unsaturated fatty acids [FA0103] | FA 18:2 |
| pos\_1007 | PE(14:0/22:4(7Z,10Z,13Z,16Z)) | LMGP02010421 | PE(14:0/22:4(7Z,10Z,13Z,16Z)) | 1-tetradecanoyl-2-(7Z,10Z,13Z,16Z-docosatetraenoyl)-glycero-3-phosphoethanolamine | PE(36:4); PE(14:0\_22:4) | Glycerophospholipids [GP] | Glycerophosphoethanolamines [GP02] | Diacylglycerophosphoethanolamines [GP0201] | PE 36:4 |
| pos\_101 | 8Z,11Z,14Z-heptadecatrienal | LMFA06000111 | 8Z,11Z,14Z-heptadecatrienal | 8Z,11Z,14Z-heptadecatrienal | - | Fatty Acyls [FA] | Fatty aldehydes [FA06] | - | FAL 17:3 |
| pos\_1017 | PE(15:0/18:4(6Z,9Z,12Z,15Z)) | LMGP02010461 | PE(15:0/18:4(6Z,9Z,12Z,15Z)) | 1-pentadecanoyl-2-(6Z,9Z,12Z,15Z-octadecatetraenoyl)-glycero-3-phosphoethanolamine | PE(33:4); PE(15:0\_18:4) | Glycerophospholipids [GP] | Glycerophosphoethanolamines [GP02] | Diacylglycerophosphoethanolamines [GP0201] | PE 33:4 |
| pos\_1030 | 2-Hydroxymyristic acid | LMFA01050484 | 2-Hydroxymyristic acid | 2-hydroxytetradecanoic acid | - | Fatty Acyls [FA] | Fatty Acids and Conjugates [FA01] | Hydroxy fatty acids [FA0105] | FA 14:0;O |
| pos\_1033 | Xanthomicrol | LMPK12111447 | Xanthomicrol | - | - | Polyketides [PK] | Flavonoids [PK12] | Flavones and Flavonols [PK1211] | - |
| pos\_1038 | Tacrolimus | LMPK04000003 | Tacrolimus | - | - | Polyketides [PK] | Macrolides and lactone polyketides [PK04] | - | - |
| pos\_1050 | Brassinolide | LMST01140001 | Brassinolide | 2alpha,3alpha,22R,23R-tetrahydroxy-6,7-seco-5-campestano-6,7-lactone | 2alpha-3alpha,22R,23R-tetrahydroxy-24-methyl-6,7-s-5alpha-cholestano-6,7-lactone | Sterol Lipids [ST] | Sterols [ST01] | Brassinolides and derivatives [ST0114] | ST 28:1;O6 |
| pos\_1055 | 7alpha,12alpha,26-trihydroxycholest-4-en-3-one | LMST04030158 | 7alpha,12alpha,26-trihydroxycholest-4-en-3-one | 7alpha,12alpha,26-trihydroxycholest-4-en-3-one | 4-cholesten-7alpha,12alpha,26-triol-3-one | Sterol Lipids [ST] | Bile acids and derivatives [ST04] | C27 bile acids, alcohols, and derivatives [ST0403] | ST 27:2;O4 |
| pos\_106 | (+)-4-Isocedren-15-al | LMPR0103690004 | (+)-4-Isocedren-15-al | - | - | Prenol Lipids [PR] | Isoprenoids [PR01] | C15 isoprenoids (sesquiterpenes) [PR0103] | - |
| pos\_1067 | PE(22:0/24:1(15Z)) | LMGP02010292 | PE(22:0/24:1(15Z)) | 1-docosanoyl-2-(15Z-tetracosenoyl)-sn-glycero-3-phosphoethanolamine | 15-Tetracosenoic acid, 1-[[[(2-aminoethoxy)hydroxyphosphinyl]oxy]methyl]-2-[(1-oxodocosyl)oxy]ethyl ester, [R-(Z)]- (9CI); PE(22:0/24:1); PE(46:1); PE(22:0\_24:1) | Glycerophospholipids [GP] | Glycerophosphoethanolamines [GP02] | Diacylglycerophosphoethanolamines [GP0201] | PE 46:1 |
| pos\_107 | Isomonospermoside | LMPK12140066 | Isomonospermoside | - | - | Polyketides [PK] | Flavonoids [PK12] | Flavanones [PK1214] | - |
| pos\_1077 | Flavoxanthin | LMPR01070271 | Flavoxanthin | - | - | Prenol Lipids [PR] | Isoprenoids [PR01] | C40 isoprenoids (tetraterpenes) [PR0107] | - |
| pos\_1079 | Cer(d18:1/14:0) | LMSP02010001 | Cer(d18:1/14:0) | N-(tetradecanoyl)-sphing-4-enine | C14 Cer; N-(tetradecanoyl)-ceramide; N-(myristoyl)-ceramide; Cer[NS] | Sphingolipids [SP] | Ceramides [SP02] | N-acylsphingosines (ceramides) [SP0201] | Cer 32:1;O2 |
| pos\_1096 | 20-oxo-leukotriene B4 | LMFA03020064 | 20-oxo-leukotriene B4 | (5S,6Z,8E,10E,12R,14Z)-5,12-dihydroxy-20-oxoicosa-6,8,10,14-tetraenoic acid | - | Fatty Acyls [FA] | Eicosanoids [FA03] | Leukotrienes [FA0302] | FA 20:5;O3 |
| pos\_110 | 4,6-heptadienal | LMFA06000025 | 4,6-heptadienal | 4,6-heptadienal | - | Fatty Acyls [FA] | Fatty aldehydes [FA06] | - | FAL 7:2 |
| pos\_1101 | Licorice glycoside D1 | LMPK12140028 | Licorice glycoside D1 | (2R)-7,4'-Dihydroxyflavanone 4'-[4-p-coumaroylapiosyl-(1->2)-glucoside] | - | Polyketides [PK] | Flavonoids [PK12] | Flavanones [PK1214] | - |
| pos\_1104 | Doxycycline | LMPK07000001 | Doxycycline | - | - | Polyketides [PK] | Linear tetracyclines [PK07] | - | - |
| pos\_1109 | Digitoxin | LMST01120018 | digitoxin | 3beta-[2,6-dideoxy-beta-D-ribo-hexopyranosyl-(1-4)-2,6-dideoxy-beta-D-ribo-hexopyranosyl-(1-4)-2,6-dideoxy-beta-D-ribo-hexopyranosyloxy]-14-hydroxy-5beta-card-20(22)-enolide | Crystodigin (TN); Digitoxin; Digitoxoside | Sterol Lipids [ST] | Sterols [ST01] | Cardanolides and derivatives [ST0112] | - |
| pos\_111 | PE(16:0/0:0) | LMGP02050002 | PE(16:0/0:0) | 1-hexadecanoyl-sn-glycero-3-phosphoethanolamine | LPE(16:0/0:0); 16:0 LYSO-PE; 1-palmitoyl-phosphatidylethenolamine; Hexadecanoic acid, 3-[[(2-aminoethoxy)hydroxyphosphinyl]oxy]-2-hydroxypropyl ester, (R)-; 1-Palmitoyl-sn-glycerol-3-L-alpha-phosphorylethanolamine; 1-Palmitoyl-sn-glycerophosphatidylethanolamine; LPE(16:0); LPE(16:0) | Glycerophospholipids [GP] | Glycerophosphoethanolamines [GP02] | Monoacylglycerophosphoethanolamines [GP0205] | LPE 16:0 |
| pos\_1114 | N-Docosahexaenoyl phenylalanine | LMFA08020094 | N-docosahexaenoyl phenylalanine | N-(4Z,7Z,10Z,12E,16Z,19Z-docosahexaenoyl)-phenylalanine | - | Fatty Acyls [FA] | Fatty amides [FA08] | N-acyl amines [FA0802] | NA 31:11;O2 |
| pos\_112 | Hyacinthin | LMPK12010139 | Hyacinthin | Cyanidin 3-(6-O-p-coumarylglucoside) | - | Polyketides [PK] | Flavonoids [PK12] | Anthocyanidins [PK1201] | - |
| pos\_1124 | Butenylcarnitine | LMFA07070053 | Butenylcarnitine | (3S)-3-[(2E)-but-2-enoyloxy]-4-(trimethylazaniumyl)butanoate | - | Fatty Acyls [FA] | Fatty esters [FA07] | Fatty acyl carnitines [FA0707] | CAR 4:1 |
| pos\_1125 | PS(18:1(9Z)/18:2(9Z,12Z)) | LMGP03010958 | PS(18:1(9Z)/18:2(9Z,12Z)) | 1-(9Z-octadecenoyl)-2-(9Z,12Z-octadecadienoyl)-glycero-3-phosphoserine | PS(36:3); PS(18:1\_18:2) | Glycerophospholipids [GP] | Glycerophosphoserines [GP03] | Diacylglycerophosphoserines [GP0301] | PS 36:3 |
| pos\_114 | 1-Hydroxy-2-(beta-D-glucosyloxy)-9,10-anthraquinone | LMFA13010061 | 1-hydroxy-2-(beta-D-glucosyloxy)-9,10-anthraquinone | 1-hydroxy-2-(beta-D-glucopyranosyloxy)-9,10-anthraquinone | 1,2-dihydroxy-9,10-anthraquinone 2-O-beta-D-glucopyranoside;1-hydroxy-2-(beta-D-glucosyloxy)-9,10-anthraquinone;1-hydroxy-9,10-dioxo-9,10-dihydroanthracen-2-yl beta-D-glucopyranoside;2-(beta-D-glucopyranosyloxy)-1-hydroxy-9,10-anthracenedione;2-alizarin-beta-D-glucoside;alizarin-2-beta-D-glucopyranoside | Fatty Acyls [FA] | Fatty acyl glycosides [FA13] | Fatty acyl glycosides of mono- and disaccharides [FA1301] | - |
| pos\_1143 | PE(15:0/20:2(11Z,14Z)) | LMGP02010465 | PE(15:0/20:2(11Z,14Z)) | 1-pentadecanoyl-2-(11Z,14Z-eicosadienoyl)-glycero-3-phosphoethanolamine | PE(35:2); PE(15:0\_20:2) | Glycerophospholipids [GP] | Glycerophosphoethanolamines [GP02] | Diacylglycerophosphoethanolamines [GP0201] | PE 35:2 |
| pos\_1146 | Norethindrone | LMST02030097 | Norethindrone | 17alpha-hydroxy-19-norpregn-4-en-20-yn-3-one | - | Sterol Lipids [ST] | Steroids [ST02] | C21 steroids (gluco/mineralocorticoids, progestogins) and derivatives [ST0203] | ST 20:4;O2 |
| pos\_1148 | Corticosterone | LMST02030186 | corticosterone | 11beta,21-dihydroxypregn-4-ene-3,20-dione | (11beta)-11,21-dihydroxypregn-4-ene-3,20-dione; 11beta,21-Dihydroxy-4-pregnene-3,20-dione; 11beta,21-dihydroxyprogesterone; 17-deoxycortisol; CORTICOSTERONE; Corticosterone; Kendall's compound B; Reichstein's substance H; corticosterone | Sterol Lipids [ST] | Steroids [ST02] | C21 steroids (gluco/mineralocorticoids, progestogins) and derivatives [ST0203] | ST 21:3;O4 |
| pos\_115 | PA(P-16:0/14:0) | LMGP10030003 | PA(P-16:0/14:0) | 1-(1Z-hexadecenyl)-2-tetradecanoyl-glycero-3-phosphate | PA(P-30:0); PA(P-16:0/14:0) | Glycerophospholipids [GP] | Glycerophosphates [GP10] | 1-(1Z-alkenyl),2-acylglycerophosphates [GP1003] | PA O-30:1 |
| pos\_1150 | 1-Acetoxy-2-hydroxy-16-heptadecen-4-one | LMFA05000594 | 1-Acetoxy-2-hydroxy-16-heptadecen-4-one | 2-hydroxy-4-oxoheptadec-16-en-1-yl acetate | - | Fatty Acyls [FA] | Fatty alcohols [FA05] | - | FOH 19:3;O3 |
| pos\_1155 | 5b-Cholestane-3a,7a,12a,23S,25-pentol | LMST01010241 | - | 5b-Cholestane-3a,7a,12a,23S,25-pentol | - | Sterol Lipids [ST] | Sterols [ST01] | Cholesterol and derivatives [ST0101] | ST 27:0;O5 |
| pos\_1158 | Withanolide A | LMST01160019 | Withanolide A | 15-[1-(4,5-dimethyl-6-oxo-3,6-dihydro-2H-pyran-2-yl)-1-hydroxyethyl]-5-hydroxy-10,14-dimethyl-3-oxapentacyclo[9.7.0.0^{2,4}.0^{5,10}.0^{14,18}]octadec-7-en-9-one | - | Sterol Lipids [ST] | Sterols [ST01] | Withanolides and derivatives [ST0116] | ST 28:6;O6 |
| pos\_116 | Isobutyl 2-furanpropionate | LMFA07010915 | Isobutyl 2-furanpropionate | 2-methylpropyl 3-(furan-2-yl)propanoate | - | Fatty Acyls [FA] | Fatty esters [FA07] | Wax monoesters [FA0701] | WE 11:3;O |
| pos\_1161 | PS(18:0/18:3(9Z,12Z,15Z)) | LMGP03010316 | PS(18:0/18:3(9Z,12Z,15Z)) | 1-octadecanoyl-2-(9Z,12Z,15Z-octadecatrienoyl)-glycero-3-phosphoserine | PS(36:3); PS(18:0\_18:3) | Glycerophospholipids [GP] | Glycerophosphoserines [GP03] | Diacylglycerophosphoserines [GP0301] | PS 36:3 |
| pos\_1168 | PA(16:0/18:2(9Z,12Z)) | LMGP10010023 | PA(16:0/18:2(9Z,12Z)) | 1-hexadecanoyl-2-(9Z,12Z-octadecadienoyl)-sn-glycero-3-phosphate | 9,12-Octadecadienoic acid (Z,Z)-, 1-[[(1-oxohexadecyl)oxy]methyl]-2-(phosphonooxy)ethyl ester, (R)-; 1-Palmitoyl-2-linoleoyl-sn-glycero-3-phosphate; PA(16:0/18:2); PA(34:2); PA(16:0\_18:2) | Glycerophospholipids [GP] | Glycerophosphates [GP10] | Diacylglycerophosphates [GP1001] | PA 34:2 |
| pos\_1169 | Testosterone | LMST02020002 | Testosterone | 17beta-hydroxyandrost-4-en-3-one | - | Sterol Lipids [ST] | Steroids [ST02] | C19 steroids (androgens) and derivatives [ST0202] | ST 19:2;O2 |
| pos\_117 | 4'-METHOXYCHALCONE | LMPK12120188 | - | 4'-Methoxychalcone | - | Polyketides [PK] | Flavonoids [PK12] | Chalcones and dihydrochalcones [PK1212] | - |
| pos\_1170 | 27-Nor-5b-cholestane-3a,7a,12a,24,25-pentol | LMST01010213 | 27-Nor-cholestane-pentol | 27-Nor-5b-cholestane-3a,7a,12a,24,25-pentol | - | Sterol Lipids [ST] | Sterols [ST01] | Cholesterol and derivatives [ST0101] | ST 26:0;O5 |
| pos\_1173 | Hypusine | LMFA01050514 | Hypusine | (2S)-2-amino-6-{[(2S)-4-amino-2-hydroxybutyl]amino}hexanoic acid | - | Fatty Acyls [FA] | Fatty Acids and Conjugates [FA01] | Hydroxy fatty acids [FA0105] | - |
| pos\_1190 | Isopersin | LMFA05000670 | Isopersin | (12Z,15Z)-1-hydroxy-4-oxohenicosa-12,15-dien-2-yl acetate | - | Fatty Acyls [FA] | Fatty alcohols [FA05] | - | FOH 23:4;O3 |
| pos\_1191 | 4alpha-Carboxy-5alpha-cholesta-8-en-3beta-ol | LMST01010228 | - | 4alpha-carboxy-5alpha-cholesta-8-en-3beta-ol | - | Sterol Lipids [ST] | Sterols [ST01] | Cholesterol and derivatives [ST0101] | ST 28:2;O3 |
| pos\_1193 | Glabrene | LMPK12080060 | Glabrene | - | - | Polyketides [PK] | Flavonoids [PK12] | Isoflavans [PK1208] | - |
| pos\_1204 | Androsterone | LMST02020001 | Androsterone | 3alpha-hydroxy-5alpha-androstan-17-one | Androsterone | Sterol Lipids [ST] | Steroids [ST02] | C19 steroids (androgens) and derivatives [ST0202] | ST 19:1;O2 |
| pos\_1207 | PC(18:4(6Z,9Z,12Z,15Z)/22:6(4Z,7Z,10Z,13Z,16Z,19Z)) | LMGP01011730 | PC(18:4(6Z,9Z,12Z,15Z)/22:6(4Z,7Z,10Z,13Z,16Z,19Z)) | 1-(6Z,9Z,12Z,15Z-octadecatetraenoyl)-2-(4Z,7Z,10Z,13Z,16Z,19Z-docosahexaenoyl)-glycero-3-phosphocholine | PC(40:10); PC(18:4\_22:6) | Glycerophospholipids [GP] | Glycerophosphocholines [GP01] | Diacylglycerophosphocholines [GP0101] | PC 40:10 |
| pos\_1218 | Cholesta-4,6-dien-3-one | LMST01010059 | 4,6-cholestadienone | cholesta-4,6-dien-3-one | - | Sterol Lipids [ST] | Sterols [ST01] | Cholesterol and derivatives [ST0101] | ST 27:3;O |
| pos\_1219 | 2,4,7-Decatrienoic acid | LMFA01030962 | 2,4,7-Decatrienoic acid | (2E,4E,7E)-deca-2,4,7-trienoic acid | - | Fatty Acyls [FA] | Fatty Acids and Conjugates [FA01] | Unsaturated fatty acids [FA0103] | FA 10:3 |
| pos\_1234 | Varanic acid | LMST04030067 | Varanic acid | 3alpha,7alpha,12alpha,24-Tetrahydroxy-5beta-cholestan-26-oic acid | - | Sterol Lipids [ST] | Bile acids and derivatives [ST04] | C27 bile acids, alcohols, and derivatives [ST0403] | ST 27:1;O6 |
| pos\_1248 | Cerebroside B | LMSP05010133 | Cerebroside B | N-(2-hydroxy-hexadecanoyl)-1-beta-glucosyl-9-methyl-sphinga-4E,8E-dienine | - | Sphingolipids [SP] | Neutral glycosphingolipids [SP05] | Simple Glc series [SP0501] | HexCer 35:2;O3 |
| pos\_1252 | Docosahexaenoic acid | LMFA01031176 | - | 5Z,8Z,11Z,14Z,17Z,20Z-docosahexaenoic acid | Docosahexaenoic acid; C22:6n-2,5,8,11,14,17;Docosahexaenoate;5Z,8Z,11Z,14Z,17Z,20Z-docosahexaenoate | Fatty Acyls [FA] | Fatty Acids and Conjugates [FA01] | Unsaturated fatty acids [FA0103] | FA 22:6 |
| pos\_1253 | Prostaglandin H1 | LMFA03010044 | PGH1 | 9S,11R-epidioxy-15S-hydroxy-13E-prostaenoic acid | Prostaglandin H1 | Fatty Acyls [FA] | Eicosanoids [FA03] | Prostaglandins [FA0301] | FA 20:3;O3 |
| pos\_1256 | Dehydrophytosphingosine | LMSP01030002 | Dehydrophytosphingosine | 4R-hydroxysphing-8E-enine | 4-hydroxy-8-sphingenine | Sphingolipids [SP] | Sphingoid bases [SP01] | 4-Hydroxysphinganines (Phytosphingosines) [SP0103] | SPB 18:1;O3 |
| pos\_1257 | PE(20:1(11Z)/22:6(4Z,7Z,10Z,13Z,16Z,19Z)) | LMGP02010870 | PE(20:1(11Z)/22:6(4Z,7Z,10Z,13Z,16Z,19Z)) | 1-(11Z-eicosenoyl)-2-(4Z,7Z,10Z,13Z,16Z,19Z-docosahexaenoyl)-glycero-3-phosphoethanolamine | PE(42:7); PE(20:1\_22:6) | Glycerophospholipids [GP] | Glycerophosphoethanolamines [GP02] | Diacylglycerophosphoethanolamines [GP0201] | PE 42:7 |
| pos\_1265 | 11-Keto-beta-boswellic acid | LMPR0106180008 | 11-Keto-beta-boswellic acid | (4R)-3alpha-hydroxy-11-oxo-urs-12-en-23-oic acid | - | Prenol Lipids [PR] | Isoprenoids [PR01] | C30 isoprenoids (triterpenes) [PR0106] | - |
| pos\_1270 | PC(14:1(9Z)/22:5(4Z,7Z,10Z,13Z,16Z)) | LMGP01012134 | PC(14:1(9Z)/22:5(4Z,7Z,10Z,13Z,16Z)) | 1-(9Z-tetradecenoyl)-2-(4Z,7Z,10Z,13Z,16Z-docosapentaenoyl)-sn-glycero-3-phosphocholine | PC(36:6); PC(14:1\_22:5) | Glycerophospholipids [GP] | Glycerophosphocholines [GP01] | Diacylglycerophosphocholines [GP0101] | PC 36:6 |
| pos\_1282 | Dolichyl diphosphate | LMPR03090023 | dolichyl diphosphate | alpha-(3-methylbut-2-en-1-yl)-omega-(4-{[hydroxy(phosphonooxy)phosphoryl]oxy}-2-methylbutyl)poly[(2E)-2-methylbut-2-ene-1,4-diyl] | Dolichol diphosphate; Dolichol pyrophosphate; Dolichyl diphosphate; Dolichyl pyrophosphate | Prenol Lipids [PR] | Polyprenols [PR03] | Dolichol diphosphates [PR0309] | - |
| pos\_1289 | Licorisoflavan A | LMPK12080046 | 7-O-Methyllicoricidin | (+)-2',4'-Dihydroxy-5,7-dimethoxy-6,3'-diprenylisoflavan | Licorisoflavan A | Polyketides [PK] | Flavonoids [PK12] | Isoflavans [PK1208] | - |
| pos\_129 | Gibberellin A24 | LMPR0104170018 | Gibberellin A24 | - | - | Prenol Lipids [PR] | Isoprenoids [PR01] | C20 isoprenoids (diterpenes) [PR0104] | - |
| pos\_1291 | 3-Methyl-5-pentyl-2-furanundecanoic acid | LMFA01150013 | 11-(3-methyl-5-pentylfuran-2-yl)-undecanoic acid | 11-(5-pentyl-3-methylfuran-2-yl)-undecanoic acid | 11M5; MeF(11,5); 3-methyl-5-pentyl-2-furanundecanoic acid | Fatty Acyls [FA] | Fatty Acids and Conjugates [FA01] | Heterocyclic fatty acids [FA0115] | FA 21:3;O |
| pos\_1295 | PE(20:3(8Z,11Z,14Z)/22:0) | LMGP02010928 | PE(20:3(8Z,11Z,14Z)/22:0) | 1-(8Z,11Z,14Z-eicosatrienoyl)-2-docosanoyl-glycero-3-phosphoethanolamine | PE(42:3); PE(20:3\_22:0) | Glycerophospholipids [GP] | Glycerophosphoethanolamines [GP02] | Diacylglycerophosphoethanolamines [GP0201] | PE 42:3 |
| pos\_130 | Lecanoric acid | LMPK13080001 | Lecanoric acid | - | - | Polyketides [PK] | Aromatic polyketides [PK13] | Depsides and depsidones [PK1308] | - |
| pos\_1301 | PS(14:1(9Z)/18:2(9Z,12Z)) | LMGP03010121 | PS(14:1(9Z)/18:2(9Z,12Z)) | 1-(9Z-tetradecenoyl)-2-(9Z,12Z-octadecadienoyl)-glycero-3-phosphoserine | PS(32:3); PS(14:1\_18:2) | Glycerophospholipids [GP] | Glycerophosphoserines [GP03] | Diacylglycerophosphoserines [GP0301] | PS 32:3 |
| pos\_1311 | 1-Deoxy-D-glucitol | LMFA05000598 | 1-Deoxy-D-glucitol | hexane-1,2,3,4,5-pentol | - | Fatty Acyls [FA] | Fatty alcohols [FA05] | - | FOH 6:0;O4 |
| pos\_1312 | 8-Hydroxyluteolin 4'-methyl ether 8-glucuronide | LMPK12111385 | 8-Hydroxyluteolin 4'-methyl ether 8-glucuronide | - | - | Polyketides [PK] | Flavonoids [PK12] | Flavones and Flavonols [PK1211] | - |
| pos\_1314 | Panaxydol | LMFA05000028 | Panaxydol | 9,10-epoxy-1-heptadecen-4,6-diyn-3-ol | 9,10-epoxy-heptadeca-1-en-4,6-diyn-3-ol | Fatty Acyls [FA] | Fatty alcohols [FA05] | - | FOH 17:6;O |
| pos\_1334 | 1,24-Tetracosanediol | LMFA05000580 | 1,24-Tetracosanediol | tetracosane-1,24-diol | - | Fatty Acyls [FA] | Fatty alcohols [FA05] | - | FOH 24:0;O |
| pos\_1339 | PE(18:3(6Z,9Z,12Z)/20:5(5Z,8Z,11Z,14Z,17Z)) | LMGP02010703 | PE(18:3(6Z,9Z,12Z)/20:5(5Z,8Z,11Z,14Z,17Z)) | 1-(6Z,9Z,12Z-octadecatrienoyl)-2-(5Z,8Z,11Z,14Z,17Z-eicosapentaenoyl)-glycero-3-phosphoethanolamine | PE(38:8); PE(18:3\_20:5) | Glycerophospholipids [GP] | Glycerophosphoethanolamines [GP02] | Diacylglycerophosphoethanolamines [GP0201] | PE 38:8 |
| pos\_1355 | Stearic acid | LMFA01010018 | Stearic acid | octadecanoic acid | Cetylacetic acid; Stearate; n-Octadecanoic acid; Octadecanoate; C18:0; Lactaric acid; Talgic acid; Bassinic acid; Stearophanic acid | Fatty Acyls [FA] | Fatty Acids and Conjugates [FA01] | Straight chain fatty acids [FA0101] | FA 18:0 |
| pos\_1383 | 3-Hexaprenyl-4-hydroxybenzoic acid | LMPR02010045 | - | 3-Hexaprenyl-4-hydroxybenzoic acid | - | Prenol Lipids [PR] | Quinones and hydroquinones [PR02] | Ubiquinones [PR0201] | - |
| pos\_14 | Decanoic acid | LMFA01010010 | Capric acid | Decanoic acid | C10:0; Caprinic acid; Decoic acid; Decylic acid | Fatty Acyls [FA] | Fatty Acids and Conjugates [FA01] | Straight chain fatty acids [FA0101] | FA 10:0 |
| pos\_1406 | Patuletin 3-gentiobioside | LMPK12112893 | Patuletin 3-gentiobioside | - | - | Polyketides [PK] | Flavonoids [PK12] | Flavones and Flavonols [PK1211] | - |
| pos\_1407 | 21-Deoxycortisol | LMST02030195 | 21-Deoxycortisol | 11beta,17alpha-dihydroxypregn-4-ene-3,20-dione | - | Sterol Lipids [ST] | Steroids [ST02] | C21 steroids (gluco/mineralocorticoids, progestogins) and derivatives [ST0203] | ST 21:3;O4 |
| pos\_142 | Kazinol E | LMPK12020237 | Kazinol E | - | - | Polyketides [PK] | Flavonoids [PK12] | Flavans, Flavanols and Leucoanthocyanidins [PK1202] | - |
| pos\_1423 | PI(16:0/20:2(11Z,14Z)) | LMGP06010165 | PI(16:0/20:2(11Z,14Z)) | 1-hexadecanoyl-2-(11Z,14Z-eicosadienoyl)-glycero-3-phospho-(1'-myo-inositol) | PI(36:2); PI(16:0\_20:2) | Glycerophospholipids [GP] | Glycerophosphoinositols [GP06] | Diacylglycerophosphoinositols [GP0601] | PI 36:2 |
| pos\_1430 | PC(20:4(5Z,8Z,11Z,14Z)/18:0) | LMGP01011907 | PC(20:4(5Z,8Z,11Z,14Z)/18:0) | 1-(5Z,8Z,11Z,14Z-eicosatetraenoyl)-2-octadecanoyl-glycero-3-phosphocholine | PC(38:4); PC(18:0\_20:4) | Glycerophospholipids [GP] | Glycerophosphocholines [GP01] | Diacylglycerophosphocholines [GP0101] | PC 38:4 |
| pos\_1442 | DG(16:0/18:1(11Z)/0:0) | LMGL02010307 | DG(16:0/18:1(11Z)/0:0) | 1-hexadecanoyl-2-(11Z-octadecenoyl)-sn-glycerol | DG(16:0/18:1/0:0) | Glycerolipids [GL] | Diradylglycerols [GL02] | Diacylglycerols [GL0201] | DG 34:1 |
| pos\_1447 | 3-alpha-Androstanediol glucuronide | LMST05010039 | 3-alpha-androstanediol glucuronide | - | - | Sterol Lipids [ST] | Steroid conjugates [ST05] | Glucuronides [ST0501] | ST 19:0;O2;GlcA |
| pos\_1467 | Cholic acid glucuronide | LMST05010044 | Cholic acid glucuronide | 3alpha-[(beta-D-Glucopyranuronosyl)oxy]-7alpha,12alpha-dihydroxy-5beta-cholanoic acid | - | Sterol Lipids [ST] | Steroid conjugates [ST05] | Glucuronides [ST0501] | ST 24:1;O5;GlcA |
| pos\_147 | Dehydroabietic acid | LMPR0104050005 | Dehydroabietic acid | - | - | Prenol Lipids [PR] | Isoprenoids [PR01] | C20 isoprenoids (diterpenes) [PR0104] | - |
| pos\_1477 | Resolvin D5 | LMFA04030003 | Resolvin D5 | 7S,17S-dihydroxy-4Z,8E,10Z,13Z,15E,19Z-docosahexaenoic acid | - | Fatty Acyls [FA] | Docosanoids [FA04] | Resolvin Ds [FA0403] | FA 22:6;O2 |
| pos\_148 | Manumycin A | LMFA08020185 | manumycin | (2E,4E,6R)-N-[(1S,5S,6R)-5-hydroxy-5-[(1E,3E,5E)-7-[(2-hydroxy-5-oxo-cyclopenten-1-yl)amino]-7-oxo-hepta-1,3,5-trienyl]-2-oxo-7-oxabicyclo[4.1.0]hept-3-en-3-yl]-2,4,6-trimethyl-deca-2,4-dienamide | Manumycin A; Ucfi-C; AC1O5PHE | Fatty Acyls [FA] | Fatty amides [FA08] | N-acyl amines [FA0802] | - |
| pos\_1480 | Mesterolone | LMST02020107 | Mesterolone | - | - | Sterol Lipids [ST] | Steroids [ST02] | C19 steroids (androgens) and derivatives [ST0202] | ST 20:1;O2 |
| pos\_1489 | Docosanamide | LMFA08010020 | Docosanamide | docosanamide | - | Fatty Acyls [FA] | Fatty amides [FA08] | Primary amides [FA0801] | - |
| pos\_1490 | Tetrahydropersin | LMFA05000700 | Tetrahydropersin | 2-hydroxy-4-oxohenicosyl acetate | - | Fatty Acyls [FA] | Fatty alcohols [FA05] | - | FOH 23:2;O3 |
| pos\_1499 | Flavone | LMPK12110097 | Flavone | - | - | Polyketides [PK] | Flavonoids [PK12] | Flavones and Flavonols [PK1211] | - |
| pos\_1503 | PE(22:2(13Z,16Z)/22:6(4Z,7Z,10Z,13Z,16Z,19Z)) | LMGP02011101 | PE(22:2(13Z,16Z)/22:6(4Z,7Z,10Z,13Z,16Z,19Z)) | 1-(13Z,16Z-docosadienoyl)-2-(4Z,7Z,10Z,13Z,16Z,19Z-docosahexaenoyl)-glycero-3-phosphoethanolamine | PE(44:8); PE(22:2\_22:6) | Glycerophospholipids [GP] | Glycerophosphoethanolamines [GP02] | Diacylglycerophosphoethanolamines [GP0201] | PE 44:8 |
| pos\_151 | Moronic acid | LMPR0106150002 | Moronic acid | 3-oxoolean-18-en-28-oic acid | - | Prenol Lipids [PR] | Isoprenoids [PR01] | C30 isoprenoids (triterpenes) [PR0106] | - |
| pos\_153 | Ursolic acid | LMPR0106180007 | ursolic acid | 3beta-hydroxyurs-12-en-28-oic acid | (3beta)-3-hydroxyurs-12-en-28-oic acid; Ursolic acid; malol; prunol; urson | Prenol Lipids [PR] | Isoprenoids [PR01] | C30 isoprenoids (triterpenes) [PR0106] | - |
| pos\_1554 | 2-Hydroxylauroylcarnitine | LMFA07070032 | 2-Hydroxylauroylcarnitine | 3-[(2-hydroxydodecanoyl)oxy]-4-(trimethylazaniumyl)butanoate | - | Fatty Acyls [FA] | Fatty esters [FA07] | Fatty acyl carnitines [FA0707] | CAR 12:0;O |
| pos\_1557 | Mesuagin | LMPK12100030 | Mesuagin | 5-Hydroxy-6'',6''-dimethyl-6-isobutyryl-4-phenylpyrano[2'',3'':7,8]coumarin | - | Polyketides [PK] | Flavonoids [PK12] | Neoflavonoids [PK1210] | - |
| pos\_157 | 3,6,9,12,15-octadecapentaenoic acid | LMFA01030359 | 3,6,9,12,15-octadecapentaenoic acid | 3,6,9,12,15-octadecapentaenoic acid | C18:5n-3,6,9,12,15 | Fatty Acyls [FA] | Fatty Acids and Conjugates [FA01] | Unsaturated fatty acids [FA0103] | FA 18:5 |
| pos\_1570 | Panaxynol | LMFA05000689 | Falcarinol | 1,9Z-heptadecadien-4,6-diyn-3R-ol | Panaxynol; CHEBI:66722; (3R,9Z)-heptadeca-1,9-dien-4,6-diyn-3-ol; 1,9-Heptadecadiene-4,6-diyn-3-ol | Fatty Acyls [FA] | Fatty alcohols [FA05] | - | FOH 17:6 |
| pos\_1577 | Octadecyl fumarate | LMFA07010979 | Octadecyl fumarate | (2Z)-4-(octadecyloxy)-4-oxobut-2-enoic acid | - | Fatty Acyls [FA] | Fatty esters [FA07] | Wax monoesters [FA0701] | WE 22:2;O2 |
| pos\_1579 | Ginsenoside Rh1 | LMPR0106080017 | Ginsenoside Rh1 | 3beta,12beta,20-trihydroxydammar-24-en-6alpha-yl-beta-D-glucopyranoside | 20(S)-Ginsenoside Rh1; Prosapogenin A2; Sanchinoside Rh1 | Prenol Lipids [PR] | Isoprenoids [PR01] | C30 isoprenoids (triterpenes) [PR0106] | - |
| pos\_1590 | D-Leucic acid | LMFA01050402 | D-Leucic acid | 2R-hydroxy-4-methyl-pentanoic acid | - | Fatty Acyls [FA] | Fatty Acids and Conjugates [FA01] | Hydroxy fatty acids [FA0105] | FA 6:0;O |
| pos\_1593 | PG(16:0/18:2(9Z,12Z)) | LMGP04010983 | PG(16:0/18:2(9Z,12Z)) | 1-hexadecanoyl-2-(9Z,12Z-octadecadienoyl)-glycero-3-phospho-(1'-sn-glycerol) | PG(34:2); PG(16:0\_18:2) | Glycerophospholipids [GP] | Glycerophosphoglycerols [GP04] | Diacylglycerophosphoglycerols [GP0401] | PG 34:2 |
| pos\_1601 | Prostaglandin E1 | LMFA03010134 | PGE1 | 9-oxo-11R,15S-dihydroxy-13E-prostaenoic acid | Prostaglandin E1 | Fatty Acyls [FA] | Eicosanoids [FA03] | Prostaglandins [FA0301] | FA 20:3;O3 |
| pos\_1603 | 1-heneicosanoyl-glycero-3-phosphate | LMGP10050032 | PA(21:0/0:0) | 1-heneicosanoyl-glycero-3-phosphate | LPA(21:0) | Glycerophospholipids [GP] | Glycerophosphates [GP10] | Monoacylglycerophosphates [GP1005] | LPA 21:0 |
| pos\_1604 | PS(20:3(8Z,11Z,14Z)/18:0) | LMGP03010600 | PS(20:3(8Z,11Z,14Z)/18:0) | 1-(8Z,11Z,14Z-eicosatrienoyl)-2-octadecanoyl-glycero-3-phosphoserine | PS(38:3); PS(18:0\_20:3) | Glycerophospholipids [GP] | Glycerophosphoserines [GP03] | Diacylglycerophosphoserines [GP0301] | PS 38:3 |
| pos\_1607 | Naringenin | LMPK12140001 | Naringenin | - | - | Polyketides [PK] | Flavonoids [PK12] | Flavanones [PK1214] | - |
| pos\_1617 | 3a,20b-Pregnanediol | LMST02030198 | - | 3a,20b-Pregnanediol | - | Sterol Lipids [ST] | Steroids [ST02] | C21 steroids (gluco/mineralocorticoids, progestogins) and derivatives [ST0203] | ST 21:0;O2 |
| pos\_1623 | Nonivamide | LMFA08020317 | Nonivamide | N-vanillyl-nonamide | pelargonic acid vanillylamide; PAVA | Fatty Acyls [FA] | Fatty amides [FA08] | N-acyl amines [FA0802] | NA 17:4;O2 |
| pos\_1633 | Tetracosahexaenoic acid | LMFA01030804 | tetracosahexaenoic acid | 2E,4E,6E,8E,10E,12E-tetracosahexaenoic acid | C24:6n-12,14,16,18,20,22 | Fatty Acyls [FA] | Fatty Acids and Conjugates [FA01] | Unsaturated fatty acids [FA0103] | FA 24:6 |
| pos\_1637 | Jurubine | LMST01070002 | Jurubine | 3beta-amino-26-O-[beta-D-glucopyranosyl]-25R-furostan-22alpha,26-diol | - | Sterol Lipids [ST] | Sterols [ST01] | Furostanols and derivatives [ST0107] | - |
| pos\_1638 | Medroxyprogesterone | LMST02030176 | medroxyprogesterone | (6alpha)-17-hydroxy-6-methylpregn-4-ene-3,20-dione | 17-Hydroxy-6alpha-methyl-pregn-4-ene-3,20-dione; 17-Hydroxy-6alpha-methylprogesterone; 17alpha-Hydroxy-6alpha-methylprogesterone; 6alpha-Methyl-17alpha-hydroxyprogesterone; 6alpha-Methyl-4-pregnen-17alpha-ol-3,20-dione; Medroxyprogesteron | Sterol Lipids [ST] | Steroids [ST02] | C21 steroids (gluco/mineralocorticoids, progestogins) and derivatives [ST0203] | ST 22:3;O3 |
| pos\_1659 | 2-Propenyl cyclohexanehexanoate | LMFA07010744 | 2-Propenyl cyclohexanehexanoate | prop-2-en-1-yl 6-cyclohexylhexanoate | - | Fatty Acyls [FA] | Fatty esters [FA07] | Wax monoesters [FA0701] | WE 15:2 |
| pos\_166 | Maslinic Acid | LMPR0106180014 | Maslinic acid | 2alpha,3beta-dihydroxyurs-12-en-29-oic acid | - | Prenol Lipids [PR] | Isoprenoids [PR01] | C30 isoprenoids (triterpenes) [PR0106] | - |
| pos\_1664 | PC(15:0/15:0) | LMGP01010530 | PC(15:0/15:0) | 1,2-dipentadecanoyl-sn-glycero-3-phosphocholine | Pentadecanoin, 1,2-di-, phosphate, ester with choline, L-; 1,2-Dipentadecanoyl-sn-glycero-3-phosphocholine; L-alpha-Dipentadecanoyl phosphatidylcholine; PC(30:0); PC(15:0\_15:0) | Glycerophospholipids [GP] | Glycerophosphocholines [GP01] | Diacylglycerophosphocholines [GP0101] | PC 30:0 |
| pos\_1684 | PC(14:1(9Z)/22:1(13Z)) | LMGP01012133 | PC(14:1(9Z)/22:1(13Z)) | 1-(9Z-tetradecenoyl)-2-(13Z-docosenoyl)-sn-glycero-3-phosphocholine | PC(36:2); PC(14:1\_22:1) | Glycerophospholipids [GP] | Glycerophosphocholines [GP01] | Diacylglycerophosphocholines [GP0101] | PC 36:2 |
| pos\_1705 | 11,14,17-Eicosatrienoic acid | LMFA01030159 | 11,14,17-eicosatrienoic acid | 11,14,17-eicosatrienoic acid | 11, 14, 17-icosatrienoic acid; C20:3n-3,6,9 | Fatty Acyls [FA] | Fatty Acids and Conjugates [FA01] | Unsaturated fatty acids [FA0103] | FA 20:3 |
| pos\_1714 | Arachidonic acid | LMFA01030001 | Arachidonic acid | 5Z,8Z,11Z,14Z-eicosatetraenoic acid | 5Z,8Z,11Z,14Z-icosatetraenoic acid; AA; Arachidonate; cis-5,8,11,14-Eicosatetraenoic acid; (all-Z)-5,8,11,14-Eicosatetraenoic acid; all-cis-5,8,11,14-Eicosatetraenoic acid | Fatty Acyls [FA] | Fatty Acids and Conjugates [FA01] | Unsaturated fatty acids [FA0103] | FA 20:4 |
| pos\_1741 | SM(d18:0/26:1(17Z)) | LMSP03010025 | SM(d18:0/26:1(17Z)) | N-(17Z-hexacosenoyl)-sphinganine-1-phosphocholine | C26:1DH Sphingomyelin | Sphingolipids [SP] | Phosphosphingolipids [SP03] | Ceramide phosphocholines (sphingomyelins) [SP0301] | SM 44:1;O2 |
| pos\_1752 | Alpha-Tocotrienol | LMPR02020054 | alpha-tocotrienol | 2R,5,7,8-tetramethyl-2-[(3E,7E)-4,8,12-trimethyltrideca-3,7,11-trien-1-yl]-3,4-dihydro-2H-chromen-6-ol | alpha-tocotrienol; zeta1-tocopherol | Prenol Lipids [PR] | Quinones and hydroquinones [PR02] | Vitamin E [PR0202] | - |
| pos\_1763 | Maysin | LMPK12110512 | Maysin | - | - | Polyketides [PK] | Flavonoids [PK12] | Flavones and Flavonols [PK1211] | - |
| pos\_177 | Sorbaldehyde | LMFA06000006 | Sorbaldehyde | 2,4-hexadienal | Sorbic aldehyde | Fatty Acyls [FA] | Fatty aldehydes [FA06] | - | FAL 6:2 |
| pos\_178 | Gibberellin A15 | LMPR0104170017 | Gibberellin A15 | - | - | Prenol Lipids [PR] | Isoprenoids [PR01] | C20 isoprenoids (diterpenes) [PR0104] | - |
| pos\_1783 | Farnesyl pyrophosphate | LMPR0103010002 | Farnesyl diphosphate | Farnesyl pyrophosphate | - | Prenol Lipids [PR] | Isoprenoids [PR01] | C15 isoprenoids (sesquiterpenes) [PR0103] | - |
| pos\_1787 | Calenduloside E | LMPR0106150003 | oleanolic acid 3-O-beta-D-glucosiduronic acid | 28-hydroxy-28-oxoolean-12-en-3beta-yl beta-D-glucopyranosiduronic acid | 3-O-(beta-D-Glucopyranuronosyl)oleanolic acid; Calenduloside E; Glycoside St-E; Momordin Ib; Oleanoic acid 3-O-glucuronide; Polysciasaponin P7; Silphioside F; oleanolic acid 3-O-glucuronide | Prenol Lipids [PR] | Isoprenoids [PR01] | C30 isoprenoids (triterpenes) [PR0106] | - |
| pos\_179 | Cyclokievitone | LMPK12050493 | Cyclokievitone | - | - | Polyketides [PK] | Flavonoids [PK12] | Isoflavonoids [PK1205] | - |
| pos\_1795 | PI(16:0/16:1(9Z)) | LMGP06010881 | PI(16:0/16:1(9Z)) | 1-hexadecanoyl-2-(9Z-hexadecenoyl)-glycero-3-phospho-(1'-myo-inositol) | PI(32:1); PI(16:0\_16:1) | Glycerophospholipids [GP] | Glycerophosphoinositols [GP06] | Diacylglycerophosphoinositols [GP0601] | PI 32:1 |
| pos\_1799 | Arjunolic acid | LMPR0106150023 | Arjunolic acid | (2alpha,3beta,4alpha)-2,3,23-Trihydroxy-olean-12-en-28-oic acid | - | Prenol Lipids [PR] | Isoprenoids [PR01] | C30 isoprenoids (triterpenes) [PR0106] | - |
| pos\_180 | 2,4,7-tridecatrienal | LMFA06000076 | 2,4,7-tridecatrienal | 2,4,7-tridecatrienal | - | Fatty Acyls [FA] | Fatty aldehydes [FA06] | - | FAL 13:3 |
| pos\_1803 | N,2,3-Trimethyl-2-(1-methylethyl)butanamide | LMFA08020189 | N,2,3-Trimethyl-2-(1-methylethyl)butanamide | N,2,3-trimethyl-2-(propan-2-yl)butanamide | - | Fatty Acyls [FA] | Fatty amides [FA08] | N-acyl amines [FA0802] | NA 10:0 |
| pos\_1819 | PS(14:0/18:3(9Z,12Z,15Z)) | LMGP03010926 | PS(14:0/18:3(9Z,12Z,15Z)) | 1-tetradecanoyl-2-(9Z,12Z,15Z-octadecatrienoyl)-glycero-3-phosphoserine | PS(32:3); PS(14:0\_18:3) | Glycerophospholipids [GP] | Glycerophosphoserines [GP03] | Diacylglycerophosphoserines [GP0301] | PS 32:3 |
| pos\_1844 | Vitamin K1 | LMPR02030028 | phylloquinone | 2-methyl-3-[(2E,7R,11R)-3,7,11,15-tetramethylhexadec-2-en-1-yl]naphthalene-1,4-dione | 2-Methyl-3-phytyl-1,4-naphthoquinone; PHYLLOQUINONE; Phylloquinone; Phytonadione; Vitamin K1 | Prenol Lipids [PR] | Quinones and hydroquinones [PR02] | Vitamin K [PR0203] | - |
| pos\_1859 | Rhamnazin 3-rutinoside | LMPK12112634 | Rhamnazin 3-rutinoside | - | - | Polyketides [PK] | Flavonoids [PK12] | Flavones and Flavonols [PK1211] | - |
| pos\_186 | 4-[5]-ladderane-butanoic acid | LMFA01140011 | 4-[5]-ladderane-butanoic acid | 4-[5]-ladderane-butanoic acid | - | Fatty Acyls [FA] | Fatty Acids and Conjugates [FA01] | Carbocyclic fatty acids [FA0114] | FA 16:5 |
| pos\_1863 | 1-hexadecyl-glycero-3-phosphate | LMGP10060005 | PA(O-16:0/0:0) | 1-hexadecyl-glycero-3-phosphate | PA(O-16:0); PA(O-16:0\_0:0) | Glycerophospholipids [GP] | Glycerophosphates [GP10] | Monoalkylglycerophosphates [GP1006] | LPA O-16:0 |
| pos\_1865 | PC(18:1(9Z)/18:1(9Z)) | LMGP01010890 | PC(18:1(9Z)/18:1(9Z)) | 1,2-di-(9Z-octadecenoyl)-sn-glycero-3-phosphocholine | Dioleoyl phosphatidylcholine; DOPC; Choline phosphate, 3-ester with L-1,2-diolein;Olein, 1,2-di-, L-, dihydrogen phosphate, monoester with choline hydroxide; 1,2-Dioleoyl-L-alpha-lecithin; 1,2-dioleoyl-sn-glycero-3-phosphocholine; PC(18:1/18:1); PC(36:2); PC(18:1/18:1) | Glycerophospholipids [GP] | Glycerophosphocholines [GP01] | Diacylglycerophosphocholines [GP0101] | PC 36:2 |
| pos\_1868 | 16-Ketoestradiol | LMST02010047 | 16-Ketoestradiol | - | - | Sterol Lipids [ST] | Steroids [ST02] | C18 steroids (estrogens) and derivatives [ST0201] | ST 18:4;O3 |
| pos\_187 | 2-Hydroxyhomopterocarpin | LMPK12070065 | 2-Hydroxyhomopterocarpin | - | - | Polyketides [PK] | Flavonoids [PK12] | Pterocarpans [PK1207] | - |
| pos\_1876 | PGP(16:0/18:1(11Z)) | LMGP05010002 | PGP(16:0/18:1(11Z)) | 1-hexadecanoyl-2-(11Z-octadecenoyl)-sn-glycero-3-phospho-(1'-sn-glycerol-3'-phosphate) | PGP(16:0/18:1); PGP(34:1); PGP(16:0\_18:1) | Glycerophospholipids [GP] | Glycerophosphoglycerophosphates [GP05] | Diacylglycerophosphoglycerophosphates [GP0501] | PGP 34:1 |
| pos\_1898 | PS(18:0/20:0) | LMGP03010962 | PS(18:0/20:0) | 1-octadecanoyl-2-eicosanoyl-glycero-3-phosphoserine | PS(38:0); PS(18:0\_20:0) | Glycerophospholipids [GP] | Glycerophosphoserines [GP03] | Diacylglycerophosphoserines [GP0301] | PS 38:0 |
| pos\_1900 | Prednisone | LMST02030180 | prednisone | 17,21-dihydroxypregna-1,4-diene-3,11,20-trione | 1,2-Dehydrocortisone; 1,4-Pregnadiene-17alpha,21-diol-3,11,20-trione; 17,21-Dihydroxypregna-1,4-diene-3,11,20-trione; Prednisone | Sterol Lipids [ST] | Steroids [ST02] | C21 steroids (gluco/mineralocorticoids, progestogins) and derivatives [ST0203] | ST 21:5;O5 |
| pos\_1904 | Linoleic acid | LMFA01030120 | Linoleic acid | 9Z,12Z-octadecadienoic acid | cis-9, cis-12-octadecadienoic acid; cis,cis-Linoleic acid; cis,cis-9,12-octadecadienoic acid; Linoleate; 9Z,12Z-Linoleic acid; Telfairic acid; C18:2n-6,9; Linolic acid; Leinolic acid | Fatty Acyls [FA] | Fatty Acids and Conjugates [FA01] | Unsaturated fatty acids [FA0103] | FA 18:2 |
| pos\_191 | Pfaffic acid | LMPR0106250001 | Pfaffic acid | - | - | Prenol Lipids [PR] | Isoprenoids [PR01] | C30 isoprenoids (triterpenes) [PR0106] | - |
| pos\_1926 | PG(16:1(9Z)/18:1(9Z)) | LMGP04010902 | PG(16:1(9Z)/18:1(9Z)) | 1-(9Z-hexadecenoyl)-2-(9Z-octadecenoyl)-glycero-3-phospho-(1'-sn-glycerol) | PG(34:2); PG(16:1\_18:1) | Glycerophospholipids [GP] | Glycerophosphoglycerols [GP04] | Diacylglycerophosphoglycerols [GP0401] | PG 34:2 |
| pos\_1930 | Cyanidin 3-(6''-p-coumarylsambubioside) | LMPK12010141 | - | Cyanidin 3-(6''-p-coumarylsambubioside) | - | Polyketides [PK] | Flavonoids [PK12] | Anthocyanidins [PK1201] | - |
| pos\_1940 | Hydroxy-alpha-sanshool | LMFA08020326 | Hydroxy-alpha-sanshool | 2'-hydroxy-N-(isobutyl)-tetradeca-2E,6Z,8E,10E-tetraenamide | - | Fatty Acyls [FA] | Fatty amides [FA08] | N-acyl amines [FA0802] | NA 16:4;O |
| pos\_195 | (E,E)-3,7,11-Trimethyl-2,6,10-dodecatrienyl heptanoate | LMFA07010546 | (E,E)-3,7,11-Trimethyl-2,6,10-dodecatrienyl heptanoate | (E,E)-3,7,11-Trimethyl-2,6,10-dodecatrienyl heptanoate | WE(12:3(2E,6E,10E)(3Me,7Me,11Me)/7:0) | Fatty Acyls [FA] | Fatty esters [FA07] | Wax monoesters [FA0701] | WE 22:3 |
| pos\_1956 | Bisnorcholic acid | LMST04050005 | Dinorcholic acid | 24-dinor-3alpha,7alpha,12alpha-trihydroxy-5beta-cholan-22-oic acid | Bisnorcholic acid | Sterol Lipids [ST] | Bile acids and derivatives [ST04] | C22 bile acids, alcohols, and derivatives [ST0405] | ST 22:1;O5 |
| pos\_1963 | PE(14:0/18:3(6Z,9Z,12Z)) | LMGP02010412 | PE(14:0/18:3(6Z,9Z,12Z)) | 1-tetradecanoyl-2-(6Z,9Z,12Z-octadecatrienoyl)-glycero-3-phosphoethanolamine | PE(32:3); PE(14:0\_18:3) | Glycerophospholipids [GP] | Glycerophosphoethanolamines [GP02] | Diacylglycerophosphoethanolamines [GP0201] | PE 32:3 |
| pos\_1973 | 13-Oxo-9,11-tridecadienoic acid | LMFA01060202 | 13-Oxo-9,11-tridecadienoic acid | 13-oxo-9E,11E-tridecadienoic acid | - | Fatty Acyls [FA] | Fatty Acids and Conjugates [FA01] | Oxo fatty acids [FA0106] | FA 13:3;O |
| pos\_198 | DIOSGENIN | LMST01080037 | Diosgenin | (25R)-spirost-5-en-3beta-diol | - | Sterol Lipids [ST] | Sterols [ST01] | Spirostanols and derivatives [ST0108] | ST 27:3;O3 |
| pos\_1982 | Prostaglandin E2 | LMFA03010003 | PGE2 | 9-oxo-11R,15S-dihydroxy-5Z,13E-prostadienoic acid | Prostaglandin E2; Dinoprostone; Prostin E2 | Fatty Acyls [FA] | Eicosanoids [FA03] | Prostaglandins [FA0301] | FA 20:4;O3 |
| pos\_1983 | CerP(d18:1/16:0) | LMSP02050002 | CerP(d18:1/16:0) | N-(hexadecanoyl)-sphing-4-enine-1-phosphate | C16 CerP | Sphingolipids [SP] | Ceramides [SP02] | Ceramide 1-phosphates [SP0205] | CerP 34:1;O2 |
| pos\_199 | alpha-curcumene | LMPR0103060015 | alpha-curcumene | 1-methyl-4-[(2R)-6-methylhept-5-en-2-yl]benzene | - | Prenol Lipids [PR] | Isoprenoids [PR01] | C15 isoprenoids (sesquiterpenes) [PR0103] | - |
| pos\_200 | 10-propyl-5,9-tridecadien-1-ol | LMFA05000015 | 10-propyl-5,9-tridecadien-1-ol | 10-propyl-5,9-tridecadien-1-ol | 10-propyl-trideca-5,9-dien-1-ol | Fatty Acyls [FA] | Fatty alcohols [FA05] | - | FOH 16:2 |
| pos\_2020 | Nandrolone | LMST02010044 | nandrolone | 17beta-hydroxyestr-4-en-3-one | (17beta)-17-hydroxyestr-4-en-3-one; 17beta-hydroxy-19-nor-4-androsten-3-one; 17beta-hydroxy-4-estren-3-one; 19-Norandrostenolone; 19-Nortestosterone; 4-estren-17beta-ol-3-one; Nandrolone | Sterol Lipids [ST] | Steroids [ST02] | C18 steroids (estrogens) and derivatives [ST0201] | ST 18:2;O2 |
| pos\_2026 | 7Z,10Z-Hexadecadienoic acid | LMFA01030807 | 7Z,10Z-hexadecadienoic acid | 7Z,10Z-hexadecadienoic acid | C16:2n-6,9 | Fatty Acyls [FA] | Fatty Acids and Conjugates [FA01] | Unsaturated fatty acids [FA0103] | FA 16:2 |
| pos\_2029 | Cembrene | LMPR0104190002 | Cembrene | - | - | Prenol Lipids [PR] | Isoprenoids [PR01] | C20 isoprenoids (diterpenes) [PR0104] | - |
| pos\_203 | Malvidin | LMPK12010004 | Malvidin | - | - | Polyketides [PK] | Flavonoids [PK12] | Anthocyanidins [PK1201] | - |
| pos\_2030 | CerP(d18:0/16:0) | LMSP02050003 | CerP(d18:0/16:0) | N-(hexadecanoyl)-sphinganine-1-phosphate | C16DH CerP; C16 dihydroceramide-1-phosphate; dihydroceramide phosphate | Sphingolipids [SP] | Ceramides [SP02] | Ceramide 1-phosphates [SP0205] | CerP 34:0;O2 |
| pos\_2035 | PE(14:1(9Z)/22:2(13Z,16Z)) | LMGP02010449 | PE(14:1(9Z)/22:2(13Z,16Z)) | 1-(9Z-tetradecenoyl)-2-(13Z,16Z-docosadienoyl)-glycero-3-phosphoethanolamine | PE(36:3); PE(14:1\_22:2) | Glycerophospholipids [GP] | Glycerophosphoethanolamines [GP02] | Diacylglycerophosphoethanolamines [GP0201] | PE 36:3 |
| pos\_2043 | Jaceidin | LMPK12112999 | Jaceidin | 5,7,4'-Trihydroxy-3,6,3'-trimethoxyflavone | Quercetagetin 3,3',6-trimethyl ether | Polyketides [PK] | Flavonoids [PK12] | Flavones and Flavonols [PK1211] | - |
| pos\_2049 | 2,4-Nonadien-1-ol | LMFA05000608 | 2,4-Nonadien-1-ol | (2E,4E)-nona-2,4-dien-1-ol | - | Fatty Acyls [FA] | Fatty alcohols [FA05] | - | FOH 9:2 |
| pos\_2054 | Desmosterol | LMST01010016 | Desmosterol | cholest-5,24-dien-3beta-ol | - | Sterol Lipids [ST] | Sterols [ST01] | Cholesterol and derivatives [ST0101] | ST 27:2;O |
| pos\_2063 | 16-Oxoestrone | LMST02010048 | 16-Oxoestrone | - | - | Sterol Lipids [ST] | Steroids [ST02] | C18 steroids (estrogens) and derivatives [ST0201] | ST 18:5;O3 |
| pos\_2072 | 2-Hydroxyestradiol | LMST02010027 | 2-hydroxyestradiol | estra-1,3,5(10)-triene-2,3,17beta-triol | - | Sterol Lipids [ST] | Steroids [ST02] | C18 steroids (estrogens) and derivatives [ST0201] | ST 18:3;O3 |
| pos\_2074 | Tauro-b-muricholic acid | LMST05040012 | Tauro-b-muricholic acid | N-(3alpha,6beta,7beta-trihydroxy-5beta-cholan-24-oyl)-taurine | Tauro-b-muricholate | Sterol Lipids [ST] | Steroid conjugates [ST05] | Taurine conjugates [ST0504] | ST 24:1;O5;T |
| pos\_2080 | Isocarlinoside | LMPK12110489 | Isocarlinoside | - | - | Polyketides [PK] | Flavonoids [PK12] | Flavones and Flavonols [PK1211] | - |
| pos\_2107 | hydroxyisovaleroyl carnitine | LMFA07070073 | hydroxyisovaleroyl carnitine | 3-hydroxy-3-isovaleryloxy-4-(trimethylammonio)butyrate | - | Fatty Acyls [FA] | Fatty esters [FA07] | Fatty acyl carnitines [FA0707] | CAR 5:0;O |
| pos\_2119 | PS(14:0/14:0) | LMGP03010028 | PS(14:0/14:0) | 1,2-ditetradecanoyl-sn-glycero-3-phosphoserine | Dimyristoyl phosphatidylserine; Serine, 2,3-bis[(1-oxotetradecyl)oxy]propyl hydrogen phosphate (ester), (R)-; DL-Serine, 2,3-bis[(1-oxotetradecyl)oxy]propyl hydrogen phosphate (ester), (R); PS(28:0); PS(14:0/14:0) | Glycerophospholipids [GP] | Glycerophosphoserines [GP03] | Diacylglycerophosphoserines [GP0301] | PS 28:0 |
| pos\_2120 | Cyanidin 3-(6''-malonylglucoside) | LMPK12010135 | - | Cyanidin 3-(6''-malonylglucoside) | Cyanidin-3-O-(6-O-malonyl-beta-D-glucopyranoside) | Polyketides [PK] | Flavonoids [PK12] | Anthocyanidins [PK1201] | - |
| pos\_2122 | 6,7-dihydro-12-epi-LTB4 | LMFA03020070 | 6,7-dihydro-12-epi-LTB4 | (5R,8E,10E,12R,14Z)-5,12-dihydroxyicosa-8,10,14-trienoic acid | - | Fatty Acyls [FA] | Eicosanoids [FA03] | Leukotrienes [FA0302] | FA 20:3;O2 |
| pos\_2135 | Aldosterone 18-glucuronide | LMST05010062 | Aldosterone 18-glucuronide | (2S,3S,4S,5R,6S)-3,4,5-trihydroxy-6-{[(2S,14R,16R)-2-(2-hydroxyacetyl)-14-methyl-11-oxo-17-oxapentacyclo[14.2.1.0^{1,5}.0^{6,15}.0^{9,14}]nonadec-9-en-18-yl]oxy}oxane-2-carboxylic acid | - | Sterol Lipids [ST] | Steroid conjugates [ST05] | Glucuronides [ST0501] | ST 21:4;O5;GlcA |
| pos\_2138 | PS(16:0/18:1(9Z)) | LMGP03010024 | PS(16:0/18:1(9Z)) | 1-hexadecanoyl-2-(9Z-octadecenoyl)-sn-glycero-3-phosphoserine | L-Serine, 3-[(1-oxohexadecyl)oxy]-2-[(1-oxo-9-octadecenyl)oxy]propyl hydrogen phosphate (ester), [R-(Z)]-; 1-Palmitoyl-2-oleoyl-sn-glycero-3-phosphatidylserine; POPS; PS(16:0/18:1); PS(34:1); PS(16:0\_18:1) | Glycerophospholipids [GP] | Glycerophosphoserines [GP03] | Diacylglycerophosphoserines [GP0301] | PS 34:1 |
| pos\_2151 | PE(14:0/20:5(5Z,8Z,11Z,14Z,17Z)) | LMGP02011241 | PE(14:0/20:5(5Z,8Z,11Z,14Z,17Z)) | 1-tetradecanoyl-2-(5Z,8Z,11Z,14Z,17Z-eicosapentaenoyl)-glycero-3-phosphoethanolamine | PE(34:5); PE(14:0\_20:5) | Glycerophospholipids [GP] | Glycerophosphoethanolamines [GP02] | Diacylglycerophosphoethanolamines [GP0201] | PE 34:5 |
| pos\_2155 | 3b,16a-Dihydroxyandrostenone sulfate | LMST05020022 | 3b,16a-Dihydroxyandrostenone sulfate | 3b,16a-Dihydroxy-5-androsten-17-one 3-sulfate | - | Sterol Lipids [ST] | Steroid conjugates [ST05] | Sulfates [ST0502] | ST 19:2;O3;S |
| pos\_2159 | PE(P-16:0/15:0) | LMGP02030011 | PE(P-16:0/15:0) | 1-(1Z-hexadecenyl)-2-pentadecanoyl-glycero-3-phosphoethanolamine | PE(P-31:0); PE(P-16:0/15:0) | Glycerophospholipids [GP] | Glycerophosphoethanolamines [GP02] | 1-(1Z-alkenyl),2-acylglycerophosphoethanolamines [GP0203] | PE O-31:1 |
| pos\_2166 | Prostaglandin A2 | LMFA03010035 | PGA2 | 9-oxo-15S-hydroxy-5Z,10Z,13E-prostatrienoic acid | Prostaglandin A2 | Fatty Acyls [FA] | Eicosanoids [FA03] | Prostaglandins [FA0301] | FA 20:5;O2 |
| pos\_2175 | CDP-DG(16:0/18:1(11Z)) | LMGP13010002 | CDP-DG(16:0/18:1(11Z)) | 1-hexadecanoyl-2-(11Z-octadecenoyl)-sn-glycero-3-cytidine-5'-diphosphate | CDP-DG(16:0/18:1); CDP-DG(34:1); CDP-DG(16:0\_18:1) | Glycerophospholipids [GP] | CDP-Glycerols [GP13] | CDP-diacylglycerols [GP1301] | CDP-DG 34:1 |
| pos\_2180 | 11-Hydroxyprogesterone 11-glucuronide | LMST05010031 | 11-Hydroxyprogesterone 11-glucuronide | - | - | Sterol Lipids [ST] | Steroid conjugates [ST05] | Glucuronides [ST0501] | ST 21:3;O3;GlcA |
| pos\_219 | 3,7,11,15-tetramethyl-2E,6E,10E,14-hexadecatetraenal | LMFA06000197 | 3,7,11,15-tetramethyl-2E,6E,10E,14-hexadecatetraenal | 3,7,11,15-tetramethyl-2E,6E,10E,14-hexadecatetraenal | - | Fatty Acyls [FA] | Fatty aldehydes [FA06] | - | FAL 20:4 |
| pos\_2194 | Myricetin | LMPK12110001 | Myricetin | - | - | Polyketides [PK] | Flavonoids [PK12] | Flavones and Flavonols [PK1211] | - |
| pos\_2195 | PG(18:0/18:0) | LMGP04010038 | PG(18:0/18:0) | 1,2-dioctadecanoyl-sn-glycero-3-phospho-(1'-sn-glycerol) | Distearoyl phosphatidylglycerol; PG(36:0); PG(18:0/18:0) | Glycerophospholipids [GP] | Glycerophosphoglycerols [GP04] | Diacylglycerophosphoglycerols [GP0401] | PG 36:0 |
| pos\_22 | 6-pentadecyl Salicylic Acid | LMPK15040002 | 6-pentadecyl salicylic acid | 2-hydroxy-6-pentadecylbenzoic acid | 6-PDSA | Polyketides [PK] | Phenolic lipids [PK15] | Anacardic acids and derivatives [PK1504] | - |
| pos\_2239 | CDP-DG(16:0/22:6(4Z,7Z,10Z,13Z,16Z,19Z)) | LMGP13010011 | CDP-DG(16:0/22:6(4Z,7Z,10Z,13Z,16Z,19Z)) | [({[(2R,3R,5R)-5-(4-amino-2-oxo-1,2-dihydropyrimidin-1-yl)-3,4-dihydroxyoxolan-2-yl]methoxy}(hydroxy)phosphoryl)oxy][(2R)-2-[(4Z,7Z,10Z,13Z,16Z,19Z)-docosa-4,7,10,13,16,19-hexaenoyloxy]-3-(hexadecanoyloxy)propoxy]phosphinic acid | CDP-DG(38:6); CDP-DG(16:0\_22:6) | Glycerophospholipids [GP] | CDP-Glycerols [GP13] | CDP-diacylglycerols [GP1301] | CDP-DG 38:6 |
| pos\_225 | Grenoblone | LMPK12120597 | Grenoblone | - | - | Polyketides [PK] | Flavonoids [PK12] | Chalcones and dihydrochalcones [PK1212] | - |
| pos\_2281 | Meloside L | LMPK12110497 | Isoorientin 2''-O-glucopyranoside | - | Meloside L | Polyketides [PK] | Flavonoids [PK12] | Flavones and Flavonols [PK1211] | - |
| pos\_2298 | 11beta,20-Dihydroxy-3-oxopregn-4-en-21-oic acid | LMST02030223 | 11beta,20-Dihydroxy-3-oxopregn-4-en-21-oic acid | 11beta,20-Dihydroxy-3-oxopregn-4-en-21-oic acid | - | Sterol Lipids [ST] | Steroids [ST02] | C21 steroids (gluco/mineralocorticoids, progestogins) and derivatives [ST0203] | ST 21:3;O5 |
| pos\_2301 | Delphinidin 3-sambubioside | LMPK12010279 | - | Delphinidin 3-sambubioside | - | Polyketides [PK] | Flavonoids [PK12] | Anthocyanidins [PK1201] | - |
| pos\_2305 | PE(20:0/22:6(4Z,7Z,10Z,13Z,16Z,19Z)) | LMGP02011176 | PE(20:0/22:6(4Z,7Z,10Z,13Z,16Z,19Z)) | 1-eicosanoyl-2-(4Z,7Z,10Z,13Z,16Z,19Z-docosahexaenoyl)-glycero-3-phosphoethanolamine | PE(42:6); PE(20:0\_22:6) | Glycerophospholipids [GP] | Glycerophosphoethanolamines [GP02] | Diacylglycerophosphoethanolamines [GP0201] | PE 42:6 |
| pos\_2308 | Bassic acid | LMPR0106150025 | Bassic acid | (4R)-2beta,3beta,23-trihydroxy-oleana-5,12-dien-28-oic acid | - | Prenol Lipids [PR] | Isoprenoids [PR01] | C30 isoprenoids (triterpenes) [PR0106] | - |
| pos\_232 | 8-Hydroxygalangin | LMPK12113088 | 8-Hydroxygalangin | - | - | Polyketides [PK] | Flavonoids [PK12] | Flavones and Flavonols [PK1211] | - |
| pos\_2321 | Rebaudioside A | LMPR01040126 | Rebaudioside A | - | Truvia | Prenol Lipids [PR] | Isoprenoids [PR01] | C20 isoprenoids (diterpenes) [PR0104] | - |
| pos\_2326 | 19(20)-EpDPE | LMFA04000038 | 19(20)-EpDPE | (+/-)-19(20)-epoxy-4Z,7Z,10Z,13Z,16Z-docosapentaenoic acid | - | Fatty Acyls [FA] | Docosanoids [FA04] | Other Docosanoids [FA0400] | FA 22:6;O |
| pos\_2337 | 20-dihydroxyleukotriene B4 | LMFA03020061 | 20-dihydroxyleukotriene B4 | (5R,6Z,8E,10E,12S,14Z)-5,12,20,20-tetrahydroxyicosa-6,8,10,14-tetraenoic acid | - | Fatty Acyls [FA] | Eicosanoids [FA03] | Leukotrienes [FA0302] | FA 20:4;O4 |
| pos\_2341 | PE(15:0/16:1(9Z)) | LMGP02011235 | PE(15:0/16:1(9Z)) | 1-pentadecanoyl-2-(9Z-hexadecenoyl)-glycero-3-phosphoethanolamine | PE(31:1); PE(15:0\_16:1) | Glycerophospholipids [GP] | Glycerophosphoethanolamines [GP02] | Diacylglycerophosphoethanolamines [GP0201] | PE 31:1 |
| pos\_2353 | Quinestrol | LMST02010037 | Quinestrol | 3-o-cyclopentyl-17alpha-ethinyl-estra-1,3,5(10)-triene-3,17beta-diol | - | Sterol Lipids [ST] | Steroids [ST02] | C18 steroids (estrogens) and derivatives [ST0201] | ST 25:6;O2 |
| pos\_2361 | 4'-Hydroxy-5,7-dimethoxyflavan | LMPK12020276 | - | 4'-Hydroxy-5,7-dimethoxyflavan | - | Polyketides [PK] | Flavonoids [PK12] | Flavans, Flavanols and Leucoanthocyanidins [PK1202] | - |
| pos\_2365 | 3-Hydroxynonyl acetate | LMFA07010753 | 3-Hydroxynonyl acetate | 3-hydroxynonyl acetate | - | Fatty Acyls [FA] | Fatty esters [FA07] | Wax monoesters [FA0701] | WE 11:0;O |
| pos\_2372 | Pelargonic acid | LMFA01010009 | Pelargonic acid | nonanoic acid | Nonylic acid; C9:0; Nonoic acid | Fatty Acyls [FA] | Fatty Acids and Conjugates [FA01] | Straight chain fatty acids [FA0101] | FA 9:0 |
| pos\_2385 | Artobiloxanthone | LMPK12110939 | Artobiloxanthone | - | - | Polyketides [PK] | Flavonoids [PK12] | Flavones and Flavonols [PK1211] | - |
| pos\_2392 | 4Alpha-hydroxymethyl-5alpha-cholesta-8-en-3beta-ol | LMST01010235 | - | 4alpha-hydroxymethyl-5alpha-cholesta-8-en-3beta-ol | - | Sterol Lipids [ST] | Sterols [ST01] | Cholesterol and derivatives [ST0101] | ST 28:1;O2 |
| pos\_240 | 11,11-Difluoro-9Z-dodecenyl acetate | LMFA07010259 | 11,11-Difluoro-9Z-dodecenyl acetate | 11,11-Difluoro-9Z-dodecenyl acetate | - | Fatty Acyls [FA] | Fatty esters [FA07] | Wax monoesters [FA0701] | - |
| pos\_2466 | Delta-Tocopherol | LMPR02020066 | delta-tocopherol | (2R)-2,8-dimethyl-2-[(4R,8R)-4,8,12-trimethyltridecyl]-3,4-dihydro-2H-chromen-6-ol | - | Prenol Lipids [PR] | Quinones and hydroquinones [PR02] | Vitamin E [PR0202] | - |
| pos\_2479 | Hydroxypropionylcarnitine | LMFA07070074 | Hydroxypropionylcarnitine | (3S)-3-[(3-hydroxypropanoyl)oxy]-4-(trimethylazaniumyl)butanoate | - | Fatty Acyls [FA] | Fatty esters [FA07] | Fatty acyl carnitines [FA0707] | CAR 3:0;O |
| pos\_248 | beta-Hydroxy-2',6'-dimethoxy-3',4'-methylenedioxydihydrochalcone | LMPK12120589 | - | beta-Hydroxy-2',6'-dimethoxy-3',4'-methylenedioxydihydrochalcone | - | Polyketides [PK] | Flavonoids [PK12] | Chalcones and dihydrochalcones [PK1212] | - |
| pos\_2481 | 5alpha-Androst-16-en-3-one | LMST02020079 | 5alpha-androst-16-en-3-one | 5alpha-androst-16-en-3-one | androstenone | Sterol Lipids [ST] | Steroids [ST02] | C19 steroids (androgens) and derivatives [ST0202] | ST 19:2;O |
| pos\_25 | 9S,10R-Epoxy-6Z-octadecene | LMFA12000284 | 9S,10R-Epoxy-6Z-octadecene | 9S,10R-Epoxy-6Z-octadecene | - | Fatty Acyls [FA] | Oxygenated hydrocarbons [FA12] | - | - |
| pos\_250 | DG(18:3(9Z,12Z,15Z)/18:3(9Z,12Z,15Z)/0:0) | LMGL02010079 | DG(18:3(9Z,12Z,15Z)/18:3(9Z,12Z,15Z)/0:0) | 1,2-di-(9Z,12Z,15Z-octadecatrienoyl)-sn-glycerol | DG(18:3/18:3/0:0) | Glycerolipids [GL] | Diradylglycerols [GL02] | Diacylglycerols [GL0201] | DG 36:6 |
| pos\_2521 | CDP-DG(18:0/22:3(10Z,13Z,16Z)) | LMGP13010019 | CDP-DG(18:0/22:3(10Z,13Z,16Z)) | [({[(2R,3R,5R)-5-(4-amino-2-oxo-1,2-dihydropyrimidin-1-yl)-3,4-dihydroxyoxolan-2-yl]methoxy}(hydroxy)phosphoryl)oxy][(2R)-2-[(10Z,13Z,16Z)-docosa-10,13,16-trienoyloxy]-3-(octadecanoyloxy)propoxy]phosphinic acid | CDP-DG(40:3); CDP-DG(18:0\_22:3) | Glycerophospholipids [GP] | CDP-Glycerols [GP13] | CDP-diacylglycerols [GP1301] | CDP-DG 40:3 |
| pos\_2538 | SM(d18:1/24:0) | LMSP03010008 | SM(d18:1/24:0) | N-(tetracosanoyl)-sphing-4-enine-1-phosphocholine | C24 Sphingomyelin | Sphingolipids [SP] | Phosphosphingolipids [SP03] | Ceramide phosphocholines (sphingomyelins) [SP0301] | SM 42:1;O2 |
| pos\_2542 | Allylestrenol | LMST02030125 | Allylestrenol | 19-norpregn-4-ene-20-yn-17beta-ol | - | Sterol Lipids [ST] | Steroids [ST02] | C21 steroids (gluco/mineralocorticoids, progestogins) and derivatives [ST0203] | ST 21:2;O |
| pos\_2559 | PE(14:0/14:0) | LMGP02010352 | PE(14:0/14:0) | 1,2-ditetradecanoyl-sn-glycero-3-phosphoethanolamine | Dimyristoyl phosphatidylethanolamine; PE(28:0); PE(14:0\_14:0) | Glycerophospholipids [GP] | Glycerophosphoethanolamines [GP02] | Diacylglycerophosphoethanolamines [GP0201] | PE 28:0 |
| pos\_257 | Isognaphalin 8-butyrate | LMPK12113102 | Isognaphalin 8-butyrate | - | - | Polyketides [PK] | Flavonoids [PK12] | Flavones and Flavonols [PK1211] | - |
| pos\_2573 | Methylthiomethyl hexanoate | LMFA07010969 | Methylthiomethyl hexanoate | (methylsulfanyl)methyl hexanoate | - | Fatty Acyls [FA] | Fatty esters [FA07] | Wax monoesters [FA0701] | - |
| pos\_2596 | Sakuranetin | LMPK12140571 | Sakuranetin | - | - | Polyketides [PK] | Flavonoids [PK12] | Flavanones [PK1214] | - |
| pos\_260 | PA(18:3(6Z,9Z,12Z)/16:0) | LMGP10010370 | PA(18:3(6Z,9Z,12Z)/16:0) | 1-(6Z,9Z,12Z-octadecatrienoyl)-2-hexadecanoyl-glycero-3-phosphate | PA(34:3); PA(16:0\_18:3) | Glycerophospholipids [GP] | Glycerophosphates [GP10] | Diacylglycerophosphates [GP1001] | PA 34:3 |
| pos\_2615 | PC(20:5(5Z,8Z,11Z,14Z,17Z)/20:1(11Z)) | LMGP01011945 | PC(20:5(5Z,8Z,11Z,14Z,17Z)/20:1(11Z)) | 1-(5Z,8Z,11Z,14Z,17Z-eicosapentaenoyl)-2-(11Z-eicosenoyl)-glycero-3-phosphocholine | PC(40:6); PC(20:1\_20:5) | Glycerophospholipids [GP] | Glycerophosphocholines [GP01] | Diacylglycerophosphocholines [GP0101] | PC 40:6 |
| pos\_2626 | Allyl nonanoate | LMFA07010778 | Allyl nonanoate | prop-2-en-1-yl nonanoate | - | Fatty Acyls [FA] | Fatty esters [FA07] | Wax monoesters [FA0701] | WE 12:1 |
| pos\_2628 | Oleoylcarnitine | LMFA07070096 | O-oleoylcarnitine | 3-[(9Z)-octadec-9-enoyloxy]-4-(trimethylazaniumyl)butanoate | (9Z)-octadec-9-enoylcarnitine;3-[(9Z)-octadec-9-enoyloxy]-4-(trimethylammonio)butanoate;acylcarnitine C18:1;oleoylcarnitine | Fatty Acyls [FA] | Fatty esters [FA07] | Fatty acyl carnitines [FA0707] | CAR 18:1 |
| pos\_2632 | PI(18:2(9Z,12Z)/22:2(13Z,16Z)) | LMGP06010332 | PI(18:2(9Z,12Z)/22:2(13Z,16Z)) | 1-(9Z,12Z-octadecadienoyl)-2-(13Z,16Z-docosadienoyl)-glycero-3-phospho-(1'-myo-inositol) | PI(40:4); PI(18:2\_22:2) | Glycerophospholipids [GP] | Glycerophosphoinositols [GP06] | Diacylglycerophosphoinositols [GP0601] | PI 40:4 |
| pos\_2639 | PI(16:0/18:0) | LMGP06010878 | PI(16:0/18:0) | 1-hexadecanoyl-2-octadecanoyl-glycero-3-phospho-(1'-myo-inositol) | PI(34:0); PI(16:0\_18:0) | Glycerophospholipids [GP] | Glycerophosphoinositols [GP06] | Diacylglycerophosphoinositols [GP0601] | PI 34:0 |
| pos\_2641 | PE(16:1(9Z)/22:0) | LMGP02010535 | PE(16:1(9Z)/22:0) | 1-(9Z-hexadecenoyl)-2-docosanoyl-glycero-3-phosphoethanolamine | PE(38:1); PE(16:1\_22:0) | Glycerophospholipids [GP] | Glycerophosphoethanolamines [GP02] | Diacylglycerophosphoethanolamines [GP0201] | PE 38:1 |
| pos\_265 | 4,10-undecadiynal | LMFA06000070 | 4,10-undecadiynal | 4,10-undecadiynal | - | Fatty Acyls [FA] | Fatty aldehydes [FA06] | - | FAL 11:4 |
| pos\_2666 | 5-cis-8-cis-Tetradecadienoyl-CoA | LMFA07050266 | 5-cis-8-cis-Tetradecadienoyl-CoA | {[5-(6-amino-9H-purin-9-yl)-4-hydroxy-2-({[hydroxy({[hydroxy(3-hydroxy-2,2-dimethyl-3-{[2-({2-[(5Z,8Z)-tetradeca-5,8-dienoylsulfanyl]ethyl}carbamoyl)ethyl]carbamoyl}propoxy)phosphoryl]oxy})phosphoryl]oxy}methyl)oxolan-3-yl]oxy}phosphonic acid | - | Fatty Acyls [FA] | Fatty esters [FA07] | Fatty acyl CoAs [FA0705] | CoA 14:2 |
| pos\_2682 | Prostaglandin E3 | LMFA03010135 | PGE3 | 9-oxo-11R,15S-dihydroxy-5Z,13E,17Z-prostatrienoic acid | Prostaglandin E3 | Fatty Acyls [FA] | Eicosanoids [FA03] | Prostaglandins [FA0301] | FA 20:5;O3 |
| pos\_2683 | PS(18:3(9Z,12Z,15Z)/20:4(5Z,8Z,11Z,14Z)) | LMGP03010417 | PS(18:3(9Z,12Z,15Z)/20:4(5Z,8Z,11Z,14Z)) | 1-(9Z,12Z,15Z-octadecatrienoyl)-2-(5Z,8Z,11Z,14Z-eicosatetraenoyl)-glycero-3-phosphoserine | PS(38:7); PS(18:3\_20:4) | Glycerophospholipids [GP] | Glycerophosphoserines [GP03] | Diacylglycerophosphoserines [GP0301] | PS 38:7 |
| pos\_2687 | 4-Fumarylacetoacetic acid | LMFA01170066 | 4-fumarylacetoacetic acid | 4,6-dioxo-2E-octenedioic acid | 4-Fumarylacetoacetic acid; Fumarylacetoacetone | Fatty Acyls [FA] | Fatty Acids and Conjugates [FA01] | Dicarboxylic acids [FA0117] | FA 8:4;O4 |
| pos\_2696 | Pentacosanoic acid | LMFA01010025 | Hyenic acid | Pentacosanoic acid | C25:0 | Fatty Acyls [FA] | Fatty Acids and Conjugates [FA01] | Straight chain fatty acids [FA0101] | FA 25:0 |
| pos\_27 | Diosmin | LMPK12110814 | Luteolin 4'-methyl ether 7-rutinoside | - | 3',5,7-Trihydroxy-4'-methoxyflavone 7-rutinoside;; Diosmin | Polyketides [PK] | Flavonoids [PK12] | Flavones and Flavonols [PK1211] | - |
| pos\_270 | Lynestrenol | LMST02030127 | Lynestrenol | 19-norpregn-4-en-20-yn-17alpha-ol | - | Sterol Lipids [ST] | Steroids [ST02] | C21 steroids (gluco/mineralocorticoids, progestogins) and derivatives [ST0203] | ST 20:3;O |
| pos\_271 | 8,11,14,18-Eicosatetraynoic acid | LMFA01030692 | 8,11,14,18-Eicosatetraynoic acid | 8,11,14,18-Eicosatetraynoic acid | - | Fatty Acyls [FA] | Fatty Acids and Conjugates [FA01] | Unsaturated fatty acids [FA0103] | FA 20:6 |
| pos\_2726 | Sorbic acid | LMFA01030100 | Sorbic acid | 2E,4E-hexadienoic acid | C6:2n-2,4 | Fatty Acyls [FA] | Fatty Acids and Conjugates [FA01] | Unsaturated fatty acids [FA0103] | FA 6:2 |
| pos\_2729 | Myrcene | LMPR0102010005 | Myrcene | - | - | Prenol Lipids [PR] | Isoprenoids [PR01] | C10 isoprenoids (monoterpenes) [PR0102] | - |
| pos\_273 | N-arachidonoyl glutamine | LMFA08020126 | N-arachidonoyl glutamine | N-(5Z,8Z,11Z,14Z-eicosatetraenoyl)-glutamine | - | Fatty Acyls [FA] | Fatty amides [FA08] | N-acyl amines [FA0802] | - |
| pos\_2732 | Ethyl butanoate | LMFA07010874 | Ethyl butyrate | ethyl butanoate | - | Fatty Acyls [FA] | Fatty esters [FA07] | Wax monoesters [FA0701] | WE 6:0 |
| pos\_2735 | 7,8-Didehydrodesmosterol | LMST01010121 | 7-dehydro-desmosterol | cholest-5,7,24-trien-3beta-ol | 24-Dehydroprovitamin D3; 7,8-Didehydrodesmosterol | Sterol Lipids [ST] | Sterols [ST01] | Cholesterol and derivatives [ST0101] | ST 27:3;O |
| pos\_2741 | 3-mercaptohexyl acetate | LMFA05000540 | 3-mercaptohexyl acetate | 3-thiohexyl acetate | - | Fatty Acyls [FA] | Fatty alcohols [FA05] | - | - |
| pos\_2743 | PG(16:0/16:1(9Z)) | LMGP04010910 | PG(16:0/16:1(9Z)) | 1-hexadecanoyl-2-(9Z-hexadecenoyl)-glycero-3-phospho-(1'-sn-glycerol) | PG(32:1); PG(16:0\_16:1) | Glycerophospholipids [GP] | Glycerophosphoglycerols [GP04] | Diacylglycerophosphoglycerols [GP0401] | PG 32:1 |
| pos\_2744 | L-Palmitoylcarnitine | LMFA07070079 | L-palmitoylcarnitine | (3S)-3-hexadecanoyloxy-4-(trimethylammonio)butanoate | (3S)-3-palmitoyloxy-4-(trimethylammonio)butanoate;(S)-palmitoylcarnitine | Fatty Acyls [FA] | Fatty esters [FA07] | Fatty acyl carnitines [FA0707] | CAR 16:0 |
| pos\_2752 | PA(16:1(9Z)/16:1(9Z)) | LMGP10010969 | PA(16:1(9Z)/16:1(9Z)) | 1,2-di-(9Z-hexadecenoyl)-sn-glycero-3-phosphate | PA(32:2); PA(16:1\_16:1) | Glycerophospholipids [GP] | Glycerophosphates [GP10] | Diacylglycerophosphates [GP1001] | PA 32:2 |
| pos\_2755 | PC(14:0/16:1(9Z)) | LMGP01010485 | PC(14:0/16:1(9Z)) | 1-tetradecanoyl-2-(9Z-hexadecenoyl)-sn-glycero-3-phosphocholine | PC(14:0/16:1); PC(30:1); PC(14:0\_16:1) | Glycerophospholipids [GP] | Glycerophosphocholines [GP01] | Diacylglycerophosphocholines [GP0101] | PC 30:1 |
| pos\_2759 | L-Acetylcarnitine | LMFA07070050 | Acetyl-L-carnitine | (3R)-3-acetyloxy-4-(trimethylazaniumyl)butanoate | L-Acetylcarnitine | Fatty Acyls [FA] | Fatty esters [FA07] | Fatty acyl carnitines [FA0707] | CAR 2:0 |
| pos\_2760 | Myristoleic acid | LMFA01030051 | Myristoleic acid | 9Z-tetradecenoic acid | cis-9-tetradecenoic acid; C14:1n-5 | Fatty Acyls [FA] | Fatty Acids and Conjugates [FA01] | Unsaturated fatty acids [FA0103] | FA 14:1 |
| pos\_2761 | 3beta-hydroxy-4beta-methyl-5alpha-cholest-7-ene-4alpha-carboxylic acid | LMST01010175 | 3beta-hydroxy-4beta-methyl-5alpha-cholest-7-ene-4alpha-carboxylic acid | 3beta-hydroxy-4beta-methyl-5alpha-cholest-7-ene-4alpha-carboxylic acid | 3beta-Hydroxy-4beta-methyl-5alpha-cholest-7-ene-4alpha-carboxylate; 3beta-hydroxy-4beta-methyl-5alpha-cholest-7-ene-4alpha-carboxylic acid | Sterol Lipids [ST] | Sterols [ST01] | Cholesterol and derivatives [ST0101] | ST 29:2;O3 |
| pos\_2769 | PC(16:1(9Z)/0:0) | LMGP01050022 | PC(16:1(9Z)/0:0) | 1-(9Z-hexadecenoyl)-sn-glycero-3-phosphocholine | 1-palmitoleoyl-glycerophosphocholine; LPC(16:1); LPC(16:1) | Glycerophospholipids [GP] | Glycerophosphocholines [GP01] | Monoacylglycerophosphocholines [GP0105] | LPC 16:1 |
| pos\_2779 | CDP-DG(18:1(9Z)/22:3(10Z,13Z,16Z)) | LMGP13010035 | CDP-DG(18:1(9Z)/22:3(10Z,13Z,16Z)) | [({[(2R,3R,5R)-5-(4-amino-2-oxo-1,2-dihydropyrimidin-1-yl)-3,4-dihydroxyoxolan-2-yl]methoxy}(hydroxy)phosphoryl)oxy][(2R)-2-[(10Z,13Z,16Z)-docosa-10,13,16-trienoyloxy]-3-[(9Z)-octadec-9-enoyloxy]propoxy]phosphinic acid | CDP-DG(40:4); CDP-DG(18:1\_22:3) | Glycerophospholipids [GP] | CDP-Glycerols [GP13] | CDP-diacylglycerols [GP1301] | CDP-DG 40:4 |
| pos\_278 | Maackiaflavanone | LMPK12140487 | Maackiaflavanone | - | - | Polyketides [PK] | Flavonoids [PK12] | Flavanones [PK1214] | - |
| pos\_2782 | Delta(6)-trans,Delta(8)-cis-leukotriene B4 | LMFA03020072 | Delta(6)-trans,Delta(8)-cis-leukotriene B4 | (5S,6E,8Z,10E,12R,14Z)-5,12-dihydroxyicosa-6,8,10,14-tetraenoic acid | (5S,12R,6E,8Z,10E,14Z)-5,12-dihydroxy-6,8,10,14-eicosatetraenoic acid;(5S,6E,8Z,10E,12R,14Z)-5,12-dihydroxyeicosa-6,8,10,14-tetraenoic acid;(6E,8Z)-LTB4;5(S),12(R)-dihydroxy-6(E),8(Z),10(E),14(Z)-eicosatetraenoic acid;5(S),12(R)-dihydroxy-6(E),8(Z),10(E),14(Z)-icosatetraenoic acid;Delta(6)-trans,Delta(8)-cis-LTB4 | Fatty Acyls [FA] | Eicosanoids [FA03] | Leukotrienes [FA0302] | FA 20:4;O2 |
| pos\_2787 | PS(17:0/14:0) | LMGP03010227 | PS(17:0/14:0) | 1-heptadecanoyl-2-tetradecanoyl-glycero-3-phosphoserine | PS(31:0); PS(14:0\_17:0) | Glycerophospholipids [GP] | Glycerophosphoserines [GP03] | Diacylglycerophosphoserines [GP0301] | PS 31:0 |
| pos\_28 | Cer(d18:0/13:0) | LMSP02020020 | Cer(d18:0/13:0) | N-(tridecanoyl)-sphinganine | Cer[NdS] | Sphingolipids [SP] | Ceramides [SP02] | N-acylsphinganines (dihydroceramides) [SP0202] | Cer 31:0;O2 |
| pos\_2801 | PS(18:1(9Z)/0:0) | LMGP03050001 | PS(18:1(9Z)/0:0) | 1-(9Z-octadecenoyl)-sn-glycero-3-phosphoserine | L-Serine, 2-hydroxy-3-[(1-oxo-9-octadecenyl)oxy]propyl hydrogen phosphate (ester), [R-(Z)]-; PS(18:1/0:0); LPS(18:1) | Glycerophospholipids [GP] | Glycerophosphoserines [GP03] | Monoacylglycerophosphoserines [GP0305] | LPS 18:1 |
| pos\_2809 | PS(17:0/16:1(9Z)) | LMGP03010230 | PS(17:0/16:1(9Z)) | 1-heptadecanoyl-2-(9Z-hexadecenoyl)-glycero-3-phosphoserine | PS(33:1); PS(16:1\_17:0) | Glycerophospholipids [GP] | Glycerophosphoserines [GP03] | Diacylglycerophosphoserines [GP0301] | PS 33:1 |
| pos\_281 | 6-methyl-heptanoic acid | LMFA01020242 | Isocaprylic acid | 6-methyl-heptanoic acid | - | Fatty Acyls [FA] | Fatty Acids and Conjugates [FA01] | Branched fatty acids [FA0102] | FA 8:0 |
| pos\_2811 | Cyclosporin A | LMPK14000003 | Cyclosporin A | - | - | Polyketides [PK] | Non-ribosomal peptide/polyketide hybrids [PK14] | - | - |
| pos\_2812 | Stearidonic Acid | LMFA01030357 | Stearidonic acid | 6Z,9Z,12Z,15Z-octadecatetraenoic acid | Moroctic acid; C18:4n-3,6,9,12; Morotic acid | Fatty Acyls [FA] | Fatty Acids and Conjugates [FA01] | Unsaturated fatty acids [FA0103] | FA 18:4 |
| pos\_2818 | C16 Sphingosine | LMSP01040008 | C16 Sphingosine | Hexadecasphing-4E-enine | Hexadecasphingosine | Sphingolipids [SP] | Sphingoid bases [SP01] | Sphingoid base homologs and variants [SP0104] | SPB 16:1;O2 |
| pos\_2822 | dodecanamide | LMFA08010001 | dodecanamide | dodecanamide | - | Fatty Acyls [FA] | Fatty amides [FA08] | Primary amides [FA0801] | - |
| pos\_2823 | 5-propylideneisolongifolane | LMPR0103500002 | 5-propylideneisolongifolane | (1S,5E,8S)-2,2,7,7-tetramethyl-5-propylidenetricyclo[6.2.1.0(1,6)]undecane | 5-propylidene-2,2,7,7-tetramethyltricyclo[6.2.1.0(1,6)]undecane | Prenol Lipids [PR] | Isoprenoids [PR01] | C15 isoprenoids (sesquiterpenes) [PR0103] | - |
| pos\_2826 | 4-Nonylphenol | LMPK15010001 | Nonylphenol | 4-nonylphenol | - | Polyketides [PK] | Phenolic lipids [PK15] | Alkyl phenols and derivatives [PK1501] | - |
| pos\_283 | Acutifolane A | LMPR0103520002 | Acutifolane A | - | - | Prenol Lipids [PR] | Isoprenoids [PR01] | C15 isoprenoids (sesquiterpenes) [PR0103] | - |
| pos\_2830 | Zearalenone | LMPK04000016 | Zearalenone | - | - | Polyketides [PK] | Macrolides and lactone polyketides [PK04] | - | - |
| pos\_2832 | Ginsenoside Rg3 | LMPR0106080010 | Ginsenoside Rg3 | 12beta,20S-dihydroxydammar-24-en-3beta-yl 2-O-betaD-glucopyranosyl-beta-D-glucopyranoside | - | Prenol Lipids [PR] | Isoprenoids [PR01] | C30 isoprenoids (triterpenes) [PR0106] | - |
| pos\_2842 | 13,14-dihydro-15-keto-PGF2alpha | LMFA03010027 | 13,14-dihydro-15-keto-PGF2alpha | 9S,11S-dihydroxy-15-oxo-5Z-prostenoic acid | 13,14-dihydro-15-keto-Prostaglandin F2alpha | Fatty Acyls [FA] | Eicosanoids [FA03] | Prostaglandins [FA0301] | FA 20:3;O3 |
| pos\_2851 | Myriocin | LMSP01080020 | Myriocin | - | - | Sphingolipids [SP] | Sphingoid bases [SP01] | Sphingoid base analogs [SP0108] | - |
| pos\_2853 | alpha-Tocotrienol | LMPR02020054 | alpha-tocotrienol | 2R,5,7,8-tetramethyl-2-[(3E,7E)-4,8,12-trimethyltrideca-3,7,11-trien-1-yl]-3,4-dihydro-2H-chromen-6-ol | alpha-tocotrienol; zeta1-tocopherol | Prenol Lipids [PR] | Quinones and hydroquinones [PR02] | Vitamin E [PR0202] | - |
| pos\_288 | 3,4-Dimethyl-5-pentyl-2-furanheptanoic acid | LMFA01150027 | 7-(3,4-dimethyl-5-pentylfuran-2-yl)-heptanoic acid | 7-(5-pentyl-3,4-dimethylfuran-2-yl)-heptanoic acid | 7D5; 3,4-dimethyl-5-pentyl-2-furanheptanoic acid | Fatty Acyls [FA] | Fatty Acids and Conjugates [FA01] | Heterocyclic fatty acids [FA0115] | FA 18:3;O |
| pos\_29 | pentanamide | LMFA08010002 | pentanamide | pentanamide | - | Fatty Acyls [FA] | Fatty amides [FA08] | Primary amides [FA0801] | - |
| pos\_297 | 2',4',6',3,4-Pentahydroxy-3'-geranyl-5-prenyldihydrochalcone | LMPK12120532 | - | 2',4',6',3,4-Pentahydroxy-3'-geranyl-5-prenyldihydrochalcone | - | Polyketides [PK] | Flavonoids [PK12] | Chalcones and dihydrochalcones [PK1212] | - |
| pos\_3 | Cholic acid | LMST04010001 | Cholic acid | 3Alpha,7Alpha,12Alpha-trihydroxy-5Beta-cholan-24-oic acid | Cholic Acid | Sterol Lipids [ST] | Bile acids and derivatives [ST04] | C24 bile acids, alcohols, and derivatives [ST0401] | ST 24:1;O5 |
| pos\_301 | Cycloviolaxanthin | LMPR01070063 | Cycloviolaxanthin | - | - | Prenol Lipids [PR] | Isoprenoids [PR01] | C40 isoprenoids (tetraterpenes) [PR0107] | - |
| pos\_315 | Methylsainfuran | LMPK12160044 | Methylsainfuran | 2-(2',4'-Dimethoxyphenyl)-5-hydroxy-6-methoxybenzofuran | - | Polyketides [PK] | Flavonoids [PK12] | Other Flavonoids [PK1216] | - |
| pos\_33 | 7-Hydroxy-2-methylisoflavone | LMPK12050001 | - | 7-Hydroxy-2-methylisoflavone | - | Polyketides [PK] | Flavonoids [PK12] | Isoflavonoids [PK1205] | - |
| pos\_331 | PE(22:2(13Z,16Z)/18:4(6Z,9Z,12Z,15Z)) | LMGP02011087 | PE(22:2(13Z,16Z)/18:4(6Z,9Z,12Z,15Z)) | 1-(13Z,16Z-docosadienoyl)-2-(6Z,9Z,12Z,15Z-octadecatetraenoyl)-glycero-3-phosphoethanolamine | PE(40:6); PE(18:4\_22:2) | Glycerophospholipids [GP] | Glycerophosphoethanolamines [GP02] | Diacylglycerophosphoethanolamines [GP0201] | PE 40:6 |
| pos\_335 | 2'-Methoxy-4'-hydroxydihydrochalcone | LMPK12120444 | - | 2'-Methoxy-4'-hydroxydihydrochalcone | - | Polyketides [PK] | Flavonoids [PK12] | Chalcones and dihydrochalcones [PK1212] | - |
| pos\_338 | PG(P-16:0/20:1(11Z)) | LMGP04030019 | PG(P-16:0/20:1(11Z)) | 1-(1Z-hexadecenyl)-2-(11Z-eicosenoyl)-glycero-3-phospho-(1'-sn-glycerol) | PG(P-36:1); PG(P-16:0/20:1) | Glycerophospholipids [GP] | Glycerophosphoglycerols [GP04] | 1-(1Z-alkenyl),2-acylglycerophosphoglycerols [GP0403] | PG O-36:2 |
| pos\_34 | p-Cymene | LMPR0102090014 | p-Cymene | - | - | Prenol Lipids [PR] | Isoprenoids [PR01] | C10 isoprenoids (monoterpenes) [PR0102] | - |
| pos\_348 | 8-C-alpha-L-Arabinosylluteolin | LMPK12110472 | 8-C-alpha-L-Arabinosylluteolin | - | - | Polyketides [PK] | Flavonoids [PK12] | Flavones and Flavonols [PK1211] | - |
| pos\_350 | (23R)-23,25-dihydroxyvitamin D3 / (23R)-23,25-dihydroxycholecalciferol | LMST03020271 | (23R)-23,25-dihydroxyvitamin D3 / (23R)-23,25-dihydroxycholecalciferol | (5Z,7E)-(3S,23R)-9,10-seco-5,7,10(19)-cholestatriene-3,23,25-triol | - | Sterol Lipids [ST] | Secosteroids [ST03] | Vitamin D3 and derivatives [ST0302] | - |
| pos\_359 | beta-Sitostenone | LMST01040249 | beta-Sitostenone | (2R,15R)-14-[(2R,5R)-5-ethyl-6-methylheptan-2-yl]-2,15-dimethyltetracyclo[8.7.0.0^{2,7}.0^{11,15}]heptadec-6-en-5-one | - | Sterol Lipids [ST] | Sterols [ST01] | Stigmasterols and C24-ethyl derivatives [ST0104] | ST 29:2;O |
| pos\_360 | Gitogenin | LMST01080010 | Gitogenin | (25R)-5alpha-spirostan-2alpha,3beta-diol | - | Sterol Lipids [ST] | Sterols [ST01] | Spirostanols and derivatives [ST0108] | ST 27:2;O4 |
| pos\_362 | CAPSAICIN | LMFA08020085 | Capsaicin | 8-Methyl-N-vanillyl-6E-nonenamide | - | Fatty Acyls [FA] | Fatty amides [FA08] | N-acyl amines [FA0802] | NA 18:5;O2 |
| pos\_366 | 12-amino-octadecanoic acid | LMFA01100008 | 12-amino-octadecanoic acid | 12-amino-octadecanoic acid | - | Fatty Acyls [FA] | Fatty Acids and Conjugates [FA01] | Amino fatty acids [FA0110] | - |
| pos\_373 | Palmitoyl Serotonin | LMFA08020302 | Palmitoyl Serotonin | N-[2-(5-hydroxy-1H-indol-3-yl)ethyl]-hexadecanamide | PA-5HT; Palmitoyl 5HT | Fatty Acyls [FA] | Fatty amides [FA08] | N-acyl amines [FA0802] | - |
| pos\_376 | Okanin 4'-O-(4'',6''-di-O-acetylglucoside) | LMPK12120175 | Okanin 4'-O-(4'',6''-di-O-acetylglucoside) | 3,4,2',3',4'-Pentahydroxychalcone 4'-O-(4'',6''-di-O-acetylglucoside) | - | Polyketides [PK] | Flavonoids [PK12] | Chalcones and dihydrochalcones [PK1212] | - |
| pos\_381 | Methyl-liquiritigenin | LMPK12140062 | Methyl-liquiritigenin | - | - | Polyketides [PK] | Flavonoids [PK12] | Flavanones [PK1214] | - |
| pos\_382 | Cycloartomunoxanthone | LMPK12111517 | Cycloartomunoxanthone | - | - | Polyketides [PK] | Flavonoids [PK12] | Flavones and Flavonols [PK1211] | - |
| pos\_389 | 2',5'-Dihydroxyflavone | LMPK12110084 | - | 2',5'-Dihydroxyflavone | - | Polyketides [PK] | Flavonoids [PK12] | Flavones and Flavonols [PK1211] | - |
| pos\_392 | Ethyl 10-undecenoate | LMFA07010850 | Ethyl 10-undecenoate | ethyl undec-10-enoate | - | Fatty Acyls [FA] | Fatty esters [FA07] | Wax monoesters [FA0701] | WE 13:1 |
| pos\_396 | 5-Cholestene-3beta,7alpha,12alpha,24,25-pentol | LMST04030082 | - | 5-Cholestene-3beta,7alpha,12alpha,24,25-pentol | - | Sterol Lipids [ST] | Bile acids and derivatives [ST04] | C27 bile acids, alcohols, and derivatives [ST0403] | ST 27:1;O5 |
| pos\_412 | 1,9Z,16-heptadecatrien-4,6-diyn-3,8-diol | LMFA05000029 | 1,9Z,16-heptadecatrien-4,6-diyn-3,8-diol | 1,9Z,16-heptadecatrien-4,6-diyn-3,8-diol | heptadeca-1,9Z,16-trien-4,6-diyn-3,8-diol | Fatty Acyls [FA] | Fatty alcohols [FA05] | - | FOH 17:7;O |
| pos\_416 | Bufalin | LMST01130001 | Bufalin | 3beta,14beta-dihydroxy-5beta-bufa-20,22-dienolide | - | Sterol Lipids [ST] | Sterols [ST01] | Bufanolides and derivatives [ST0113] | ST 24:4;O4 |
| pos\_423 | PE(18:4(6Z,9Z,12Z,15Z)/22:6(4Z,7Z,10Z,13Z,16Z,19Z)) | LMGP02010768 | PE(18:4(6Z,9Z,12Z,15Z)/22:6(4Z,7Z,10Z,13Z,16Z,19Z)) | 1-(6Z,9Z,12Z,15Z-octadecatetraenoyl)-2-(4Z,7Z,10Z,13Z,16Z,19Z-docosahexaenoyl)-glycero-3-phosphoethanolamine | PE(40:10); PE(18:4\_22:6) | Glycerophospholipids [GP] | Glycerophosphoethanolamines [GP02] | Diacylglycerophosphoethanolamines [GP0201] | PE 40:10 |
| pos\_427 | Infectocaryone | LMPK12120418 | Infectocaryone | - | - | Polyketides [PK] | Flavonoids [PK12] | Chalcones and dihydrochalcones [PK1212] | - |
| pos\_436 | (9R,13R)-1a,1b-dinor-10,11-dihydro-12-oxo-15-phytoenoic acid | LMFA02010008 | (9R,13R)-1a,1b-dinor-10,11-dihydro-12-oxo-15-phytoenoic acid | (1R,2R)-3-oxo-2-(2'Z-pentenyl)cyclopentanehexanoic acid | - | Fatty Acyls [FA] | Octadecanoids [FA02] | 12-oxophytodienoic acid metabolites [FA0201] | FA 16:3;O |
| pos\_438 | nandrolone | LMST02010044 | nandrolone | 17beta-hydroxyestr-4-en-3-one | (17beta)-17-hydroxyestr-4-en-3-one; 17beta-hydroxy-19-nor-4-androsten-3-one; 17beta-hydroxy-4-estren-3-one; 19-Norandrostenolone; 19-Nortestosterone; 4-estren-17beta-ol-3-one; Nandrolone | Sterol Lipids [ST] | Steroids [ST02] | C18 steroids (estrogens) and derivatives [ST0201] | ST 18:2;O2 |
| pos\_44 | 3-oxoglycyrrhetinic acid | LMPR0106150012 | 3-oxoglycyrrhetinic acid | 3,11-dioxoolean-12-en-30-oic acid | 3-Oxoglycyrrhetinate; 3-oxoglycyrrhetinic acid | Prenol Lipids [PR] | Isoprenoids [PR01] | C30 isoprenoids (triterpenes) [PR0106] | - |
| pos\_441 | PA(20:1(11Z)/17:0) | LMGP10010534 | PA(20:1(11Z)/17:0) | 1-(11Z-eicosenoyl)-2-heptadecanoyl-glycero-3-phosphate | PA(37:1); PA(17:0\_20:1) | Glycerophospholipids [GP] | Glycerophosphates [GP10] | Diacylglycerophosphates [GP1001] | PA 37:1 |
| pos\_450 | 9-hydroxy-hexadecan-1,16-dioic acid | LMFA01170057 | 9-hydroxy-hexadecan-1,16-dioic acid | 9-hydroxy-hexadecan-1,16-dioic acid | - | Fatty Acyls [FA] | Fatty Acids and Conjugates [FA01] | Dicarboxylic acids [FA0117] | FA 16:1;O3 |
| pos\_454 | 5,8,11-Eicosatriynoic Acid | LMFA01030696 | 5,8,11-Eicosatriynoic acid | 5,8,11-Eicosatriynoic acid | - | Fatty Acyls [FA] | Fatty Acids and Conjugates [FA01] | Unsaturated fatty acids [FA0103] | FA 20:6 |
| pos\_459 | Isopropyl tiglate | LMFA07010924 | Isopropyl tiglate | propan-2-yl (2Z)-2-methylbut-2-enoate | - | Fatty Acyls [FA] | Fatty esters [FA07] | Wax monoesters [FA0701] | WE 8:1 |
| pos\_46 | 6-Ethyl-4-methyl-3E,5E,7E-decatriene | LMFA11000053 | 6-Ethyl-4-methyl-3E,5E,7E-decatriene | 6-Ethyl-4-methyl-3E,5E,7E-decatriene | - | Fatty Acyls [FA] | Hydrocarbons [FA11] | - | - |
| pos\_463 | Jasmonic acid | LMFA02020001 | Jasmonic acid | (1R,2R)-3-oxo-2-(pent-2Z-enyl)-cyclopentaneacetic acid | (-)-Jasmonic acid; (3R,7R)-Jasmonic acid | Fatty Acyls [FA] | Octadecanoids [FA02] | Jasmonic acids [FA0202] | FA 12:3;O |
| pos\_467 | Capsanthin 3,6-epoxide | LMPR01070050 | Capsanthin 3,6-epoxide | - | - | Prenol Lipids [PR] | Isoprenoids [PR01] | C40 isoprenoids (tetraterpenes) [PR0107] | - |
| pos\_478 | Caprylic acid | LMFA01010008 | Caprylic acid | octanoic acid | Octylic acid; C8:0 | Fatty Acyls [FA] | Fatty Acids and Conjugates [FA01] | Straight chain fatty acids [FA0101] | FA 8:0 |
| pos\_48 | (Z)-2-Decene-4,6,8-triyn-1-ol | LMFA05000569 | (Z)-2-Decene-4,6,8-triyn-1-ol | (2E)-dec-2-en-4,6,8-triyn-1-ol | - | Fatty Acyls [FA] | Fatty alcohols [FA05] | - | FOH 10:7 |
| pos\_486 | Biochanin A | LMPK12050229 | Biochanin A | 5,7-Dihydroxy-4'-methoxyisoflavone | Pratensol | Polyketides [PK] | Flavonoids [PK12] | Isoflavonoids [PK1205] | - |
| pos\_49 | 5-(1-hydroxypropan-2-yl)isolongifol-5-ene | LMPR0103500003 | 5-(1-hydroxypropan-2-yl)isolongifol-5-ene | 2-[(1R,8S)-2,2,7,7-tetramethyltricyclo[6.2.1.0(1,6)]undec-5-en-5-yl]propan-1-ol | 2-(2,2,7,7-tetramethyltricyclo[6.2.1.0(1,6)]undec-5-en-5-yl)propan-1-ol | Prenol Lipids [PR] | Isoprenoids [PR01] | C15 isoprenoids (sesquiterpenes) [PR0103] | - |
| pos\_494 | Methyl nonanoate | LMFA07010963 | Methyl nonanoate | methyl nonanoate | - | Fatty Acyls [FA] | Fatty esters [FA07] | Wax monoesters [FA0701] | WE 10:0 |
| pos\_499 | Oleic acid | LMFA01030002 | Oleic acid | 9Z-octadecenoic acid | 9-octadecylenic acid; cis-9-octadecenoic acid; cis-Oleic acid; cis-Oleate; Oleate; Elaic acid; Elaidoic acid; C18:1n-9; Rapinic acid | Fatty Acyls [FA] | Fatty Acids and Conjugates [FA01] | Unsaturated fatty acids [FA0103] | FA 18:1 |
| pos\_50 | 8(S)-HETrE | LMFA03050029 | 8(S)-HETrE | 8-hydroxyeicosa-9,11,14-trienoic acid | - | Fatty Acyls [FA] | Eicosanoids [FA03] | Hydroxy/hydroperoxyeicosatrienoic acids [FA0305] | FA 20:3;O |
| pos\_507 | Androstanedione | LMST02020085 | - | 5alpha-androstane-3,17-dione | 5alpha-Androstane-3,17-dione; 5alpha-androstane-3,17-dione; Androstanedione | Sterol Lipids [ST] | Steroids [ST02] | C19 steroids (androgens) and derivatives [ST0202] | ST 19:2;O2 |
| pos\_513 | N-Oleoyl GABA | LMFA08020104 | N-oleoyl GABA | N-(9Z-octadecenoyl)-gamma-aminobutyric acid | - | Fatty Acyls [FA] | Fatty amides [FA08] | N-acyl amines [FA0802] | NA 22:2;O2 |
| pos\_517 | 18-Oxocortisol | LMST02030194 | 18-Oxocortisol | - | - | Sterol Lipids [ST] | Steroids [ST02] | C21 steroids (gluco/mineralocorticoids, progestogins) and derivatives [ST0203] | ST 21:4;O6 |
| pos\_527 | Gingerglycolipid C | LMGL04010018 | Gingerglycolipid C | 1-(9Z-octadecenoy)l-3-O-(6'-O-alpha-D-galactosyl-beta-D-galactosyl)-sn-glycerol | - | Glycerolipids [GL] | Glycosylmonoradylglycerols [GL04] | Glycosylmonoacylglycerols [GL0401] | DGMG 18:1 |
| pos\_541 | Tridecanal | LMFA06000074 | tridecanal | tridecanal | - | Fatty Acyls [FA] | Fatty aldehydes [FA06] | - | FAL 13:0 |
| pos\_549 | PG(18:0/22:6(4Z,7Z,10Z,13Z,16Z,19Z)) | LMGP04010040 | PG(18:0/22:6(4Z,7Z,10Z,13Z,16Z,19Z)) | 1-octadecanoyl-2-(4Z,7Z,10Z,13Z,16Z,19Z-docosahexaenoyl)-sn-glycero-3-phospho-(1'-sn-glycerol) | PG(18:0/22:6); PG(40:6); PG(18:0\_22:6) | Glycerophospholipids [GP] | Glycerophosphoglycerols [GP04] | Diacylglycerophosphoglycerols [GP0401] | PG 40:6 |
| pos\_55 | iso-Debromo-laurinterol | LMPR0103150001 | iso-Debromo-laurinterol | - | - | Prenol Lipids [PR] | Isoprenoids [PR01] | C15 isoprenoids (sesquiterpenes) [PR0103] | - |
| pos\_554 | Hypogeic acid | LMFA01030808 | Hypogeic acid | 7E-hexadecenoic acid | C16:1n-9 | Fatty Acyls [FA] | Fatty Acids and Conjugates [FA01] | Unsaturated fatty acids [FA0103] | FA 16:1 |
| pos\_56 | Diosmetin | LMPK12110824 | Diosmetin | 5,7,3'-Trihydroxy-4'-methoxyflavone | 4'-Methylluteolin | Polyketides [PK] | Flavonoids [PK12] | Flavones and Flavonols [PK1211] | - |
| pos\_560 | Dodecanedioic acid | LMFA01170009 | Dodecanedioic acid | Dodecanedioic acid | - | Fatty Acyls [FA] | Fatty Acids and Conjugates [FA01] | Dicarboxylic acids [FA0117] | FA 12:1;O2 |
| pos\_562 | 11beta-Hydroxy-3,20-dioxopregn-4-en-21-oic acid | LMST02030225 | HDOPA | 11beta-Hydroxy-3,20-dioxopregn-4-en-21-oic acid | - | Sterol Lipids [ST] | Steroids [ST02] | C21 steroids (gluco/mineralocorticoids, progestogins) and derivatives [ST0203] | ST 21:4;O5 |
| pos\_563 | Propenoylcarnitine | LMFA07070104 | Propenoylcarnitine | (3S)-3-(prop-2-enoyloxy)-4-(trimethylazaniumyl)butanoate | - | Fatty Acyls [FA] | Fatty esters [FA07] | Fatty acyl carnitines [FA0707] | CAR 3:1 |
| pos\_567 | Kaempferol 3-neohesperidoside-7-(2''-p-coumaryllaminaribioside) | LMPK12111827 | Kaempferol 3-neohesperidoside-7-(2''-p-coumaryllaminaribioside) | - | - | Polyketides [PK] | Flavonoids [PK12] | Flavones and Flavonols [PK1211] | - |
| pos\_57 | 7-dehydrocholesterol | LMST01010069 | 7-dehydrocholesterol | cholesta-5,7-dien-3beta-ol | 7-DHC | Sterol Lipids [ST] | Sterols [ST01] | Cholesterol and derivatives [ST0101] | ST 27:2;O |
| pos\_577 | Prostaglandin J2 | LMFA03010019 | PGJ2 | 11-oxo-15S-hydroxy-5Z,9,13E-prostatrienoic acid | Prostaglandin J2; 8-epi-15-J2t-IsoP; 11-oxo-15S-hydroxy-5Z,9,13E-prostatrienoic acid-cyclo[8S,12R] | Fatty Acyls [FA] | Eicosanoids [FA03] | Prostaglandins [FA0301] | FA 20:5;O2 |
| pos\_581 | 1-Tridecene | LMFA11000331 | 1-Tridecene | 1-Tridecene | - | Fatty Acyls [FA] | Hydrocarbons [FA11] | - | - |
| pos\_594 | 19-Norandrosterone | LMST02010042 | 19-norandrosterone | 3alpha-hydroxy-5alpha-estran-17-one | 19-Noreoiandrosterone; 19-Noretiocholanolone | Sterol Lipids [ST] | Steroids [ST02] | C18 steroids (estrogens) and derivatives [ST0201] | ST 18:1;O2 |
| pos\_604 | Isobutyryl-L-carnitine | LMFA07070075 | Isobutyryl-L-carnitine | 3-[(2-methylpropanoyl)oxy]-4-(trimethylazaniumyl)butanoate | - | Fatty Acyls [FA] | Fatty esters [FA07] | Fatty acyl carnitines [FA0707] | CAR 4:0 |
| pos\_605 | Trifolirhizin | LMPK12070033 | (-)-Maackiain 3-O-glucoside | - | Trifolirhizin | Polyketides [PK] | Flavonoids [PK12] | Pterocarpans [PK1207] | - |
| pos\_606 | 6-Deoxocastasterone | LMST01030127 | 6-Deoxocastasterone | campestan-2alpha,3alpha,22R,23R-tetrol | - | Sterol Lipids [ST] | Sterols [ST01] | Ergosterols and C24-methyl derivatives [ST0103] | ST 28:0;O4 |
| pos\_607 | 11-Dehydro-thromboxane B2 | LMFA03030014 | 11-Dehydro-thromboxane B2 | (5E)-7-[(2R,3S,4S)-4-hydroxy-2-[(1E,3S)-3-hydroxyoct-1-en-1-yl]-6-oxooxan-3-yl]hept-5-enoic acid | - | Fatty Acyls [FA] | Eicosanoids [FA03] | Thromboxanes [FA0303] | FA 20:4;O4 |
| pos\_612 | Isorhamnetin | LMPK12110002 | Isorhamnetin | - | - | Polyketides [PK] | Flavonoids [PK12] | Flavones and Flavonols [PK1211] | - |
| pos\_618 | 11-Ketoetiocholanolone | LMST02020087 | 11-Ketoetiocholanolone | - | - | Sterol Lipids [ST] | Steroids [ST02] | C19 steroids (androgens) and derivatives [ST0202] | ST 19:2;O3 |
| pos\_630 | Linoleamide | LMFA08010008 | Linoleamide | 9Z,12Z-octadecadienamide | - | Fatty Acyls [FA] | Fatty amides [FA08] | Primary amides [FA0801] | - |
| pos\_631 | Lithocholic acid glycine conjugate | LMST05030009 | Glycolithocholic Acid | N-[(3alpha,5beta)-3-hydroxy-24-oxocholan-24-yl]-glycine | Lithocholic acid glycine conjugate | Sterol Lipids [ST] | Steroid conjugates [ST05] | Glycine conjugates [ST0503] | ST 24:1;O3;G |
| pos\_643 | 2-Hydroxy-6-pentadecylbenzoic acid | LMPK15040002 | 6-pentadecyl salicylic acid | 2-hydroxy-6-pentadecylbenzoic acid | 6-PDSA | Polyketides [PK] | Phenolic lipids [PK15] | Anacardic acids and derivatives [PK1504] | - |
| pos\_653 | 1-Heptadecene-4,6-diyne-3,9-diol | LMFA05000599 | 1-Heptadecene-4,6-diyne-3,9-diol | heptadec-1-en-4,6-diyne-3,9-diol | - | Fatty Acyls [FA] | Fatty alcohols [FA05] | - | FOH 17:5;O |
| pos\_659 | Stearidonic acid | LMFA01030357 | Stearidonic acid | 6Z,9Z,12Z,15Z-octadecatetraenoic acid | Moroctic acid; C18:4n-3,6,9,12; Morotic acid | Fatty Acyls [FA] | Fatty Acids and Conjugates [FA01] | Unsaturated fatty acids [FA0103] | FA 18:4 |
| pos\_660 | Octadecanol | LMFA05000085 | stearyl alcohol | octadecan-1-ol | 1-hydroxyoctadecane; 1-octadecanol; Stearylalkohol; n-1-octadecanol; n-octadecanol; n-octadecyl alcohol; octadecanol; stearyl alcohol | Fatty Acyls [FA] | Fatty alcohols [FA05] | - | FOH 18:0 |
| pos\_663 | Myristic acid | LMFA01010014 | Myristic acid | tetradecanoic acid | C14:0 | Fatty Acyls [FA] | Fatty Acids and Conjugates [FA01] | Straight chain fatty acids [FA0101] | FA 14:0 |
| pos\_667 | 3-Pentadecylphenol | LMPK15010004 | 3-pentadecylphenol | 3-pentadecylphenol | - | Polyketides [PK] | Phenolic lipids [PK15] | Alkyl phenols and derivatives [PK1501] | - |
| pos\_669 | Glycochenodeoxycholic acid 3-glucuronide | LMST05010048 | Glycochenodeoxycholic acid 3-glucuronide | - | - | Sterol Lipids [ST] | Steroid conjugates [ST05] | Glucuronides [ST0501] | - |
| pos\_685 | 4-Hydroxy-2,3,9-trimethoxypterocarpan | LMPK12070089 | 4-Hydroxy-2,3,9-trimethoxypterocarpan | - | - | Polyketides [PK] | Flavonoids [PK12] | Pterocarpans [PK1207] | - |
| pos\_687 | Ganoderic acid A | LMST01010391 | Ganoderic acid A | (25R)-3,11,23-trioxo-7beta,15alpha-dihydroxy-lanost-8-ene-26-oic acid | - | Sterol Lipids [ST] | Sterols [ST01] | Cholesterol and derivatives [ST0101] | ST 30:5;O7 |
| pos\_689 | N-Stearoyl phenylalanine | LMFA08020093 | N-stearoyl phenylalanine | N-octadecanoyl-phenylalanine | - | Fatty Acyls [FA] | Fatty amides [FA08] | N-acyl amines [FA0802] | NA 27:5;O2 |
| pos\_695 | Gamma-Linolenic acid | LMFA01030141 | gamma-Linolenic acid | 6Z,9Z,12Z-octadecatrienoic acid | cis-6, cis-9, cis-12-octadecatrienoic acid; C18:3n-6,9,12; GLA; Gammolenic acid; Gamolenic acid | Fatty Acyls [FA] | Fatty Acids and Conjugates [FA01] | Unsaturated fatty acids [FA0103] | FA 18:3 |
| pos\_699 | Ginsenoside C | LMPR0106080016 | Ginsenoside Rb2 | (3beta,12beta)-20-[(6-O-alpha-L-arabinopyranosyl-beta-D-glucopyranosyl)oxy]-12-hydroxydammar-24-en-3-yl 2-O-beta-D-glucopyranosyl-beta-D-glucopyranoside | Ginsenoside C | Prenol Lipids [PR] | Isoprenoids [PR01] | C30 isoprenoids (triterpenes) [PR0106] | - |
| pos\_705 | 1-(8Z,11Z,14Z-eicosatrienoyl)-glycero-3-phosphate | LMGP10050028 | PA(20:3(8Z,11Z,14Z)/0:0) | 1-(8Z,11Z,14Z-eicosatrienoyl)-glycero-3-phosphate | LPA(20:3) | Glycerophospholipids [GP] | Glycerophosphates [GP10] | Monoacylglycerophosphates [GP1005] | LPA 20:3 |
| pos\_709 | 5-Aminopentanoic acid | LMFA01100040 | 5-amino-pentanoic acid | 5-amino-pentanoic acid | 5-Aminopentanoic acid | Fatty Acyls [FA] | Fatty Acids and Conjugates [FA01] | Amino fatty acids [FA0110] | - |
| pos\_718 | PC(14:0/18:4(6Z,9Z,12Z,15Z)) | LMGP01010499 | PC(14:0/18:4(6Z,9Z,12Z,15Z)) | 1-tetradecanoyl-2-(6Z,9Z,12Z,15Z-octadecatetraenoyl)-sn-glycero-3-phosphocholine | PC(14:0/18:4); PC(32:4); PC(14:0\_18:4) | Glycerophospholipids [GP] | Glycerophosphocholines [GP01] | Diacylglycerophosphocholines [GP0101] | PC 32:4 |
| pos\_72 | 3,5,7-Trimethyl-2E,4E,6E,8E-decatetraene | LMFA11000054 | 3,5,7-Trimethyl-2E,4E,6E,8E-decatetraene | 3,5,7-Trimethyl-2E,4E,6E,8E-decatetraene | - | Fatty Acyls [FA] | Hydrocarbons [FA11] | - | - |
| pos\_723 | Leukotriene A4 | LMFA03020023 | LTA4 | 5S,6S-epoxy-7E,9E,11Z,14Z-eicosatetraenoic acid | Leukotriene A4 | Fatty Acyls [FA] | Eicosanoids [FA03] | Leukotrienes [FA0302] | FA 20:5;O |
| pos\_726 | 5,8,12-Trihydroxy-9-octadecenoic acid | LMFA02000221 | 5,8,12-TriHOME(9) | 5,8,12-trihydroxy-9-octadecenoic acid | 9-Octadecenoic acid, 5,8,12-trihydroxy- | Fatty Acyls [FA] | Octadecanoids [FA02] | Other Octadecanoids [FA0200] | FA 18:1;O3 |
| pos\_735 | Abietinol | LMPR0104050006 | abietol | abieta-7,13-dien-18-ol | Abietinol; Abietol; abietadienol; abietyl alcohol | Prenol Lipids [PR] | Isoprenoids [PR01] | C20 isoprenoids (diterpenes) [PR0104] | - |
| pos\_755 | Undecylenic acid | LMFA01030036 | Undecylenic acid | 10-undecenoic acid | 10-undecylenic acid; C11:1n-1; 10-hendecenoic acid | Fatty Acyls [FA] | Fatty Acids and Conjugates [FA01] | Unsaturated fatty acids [FA0103] | FA 11:1 |
| pos\_76 | (+)-Vulgraon B | LMPR0103490003 | (+)-Vulgraon B | (+)-3-longipinen-5-one | - | Prenol Lipids [PR] | Isoprenoids [PR01] | C15 isoprenoids (sesquiterpenes) [PR0103] | - |
| pos\_763 | PC(o-16:0/20:4(8Z,11Z,14Z,17Z)) | LMGP01020245 | PC(O-16:0/20:4(8Z,11Z,14Z,17Z)) | 1-hexadecyl-2-(8Z,11Z,14Z,17Z-eicosatetraenoyl)-sn-glycero-3-phosphocholine | PC(O-36:4); PC(O-16:0/20:4) | Glycerophospholipids [GP] | Glycerophosphocholines [GP01] | 1-alkyl,2-acylglycerophosphocholines [GP0102] | PC O-36:4 |
| pos\_769 | Cucurbitaxanthin A | LMPR01070064 | Cucurbitaxanthin A | - | - | Prenol Lipids [PR] | Isoprenoids [PR01] | C40 isoprenoids (tetraterpenes) [PR0107] | - |
| pos\_775 | 1-Acetoxy-2-hydroxy-16-heptadecyn-4-one | LMFA05000595 | 1-Acetoxy-2-hydroxy-16-heptadecyn-4-one | 2-hydroxy-4-oxoheptadec-16-yn-1-yl acetate | - | Fatty Acyls [FA] | Fatty alcohols [FA05] | - | FOH 19:4;O3 |
| pos\_779 | Neuroprotectin D1 | LMFA04040001 | Neuroprotectin D1 | 10R,17S-dihydroxy-4Z,7Z,11E,13E,15Z,19Z-docosahexaenoic acid | NPD1; Protectin D1; PD1 | Fatty Acyls [FA] | Docosanoids [FA04] | Protectins [FA0404] | FA 22:6;O2 |
| pos\_78 | Chamazulene | LMPR0103410002 | Chamazulene | - | - | Prenol Lipids [PR] | Isoprenoids [PR01] | C15 isoprenoids (sesquiterpenes) [PR0103] | - |
| pos\_783 | Limocitrol 3-glucoside | LMPK12113320 | Limocitrol 3-glucoside | - | - | Polyketides [PK] | Flavonoids [PK12] | Flavones and Flavonols [PK1211] | - |
| pos\_788 | Cucurbitacin D | LMST01010106 | Cucurbitacin D | - | - | Sterol Lipids [ST] | Sterols [ST01] | Cholesterol and derivatives [ST0101] | ST 30:5;O7 |
| pos\_8 | Taurodeoxycholic acid | LMST05040013 | Taurodeoxycholic acid | N-(3alpha,12alpha-dihydroxy-5beta-cholan-24-oyl)-taurine | Taurodeoxycholate | Sterol Lipids [ST] | Steroid conjugates [ST05] | Taurine conjugates [ST0504] | ST 24:1;O4;T |
| pos\_80 | Elongatin | LMPK12050312 | Elongatin | - | - | Polyketides [PK] | Flavonoids [PK12] | Isoflavonoids [PK1205] | - |
| pos\_801 | Gamma-linolenyl carnitine | LMFA07070112 | Gamma-linolenyl carnitine | (4S)-4-[(6Z,9Z,12Z)-octadeca-6,9,12-trienoyloxy]-4-(trimethylazaniumyl)butanoate | Gamma-linolenoyl carnitine; O-((6Z,9Z,12Z)-octadecacatrienoyl)-S-carnitine | Fatty Acyls [FA] | Fatty esters [FA07] | Fatty acyl carnitines [FA0707] | CAR 18:3 |
| pos\_802 | Narirutin | LMPK12140236 | Narirutin | - | Isonaringin; Naringenin 7-O-rutinoside | Polyketides [PK] | Flavonoids [PK12] | Flavanones [PK1214] | - |
| pos\_805 | Capric acid | LMFA01010010 | Capric acid | Decanoic acid | C10:0; Caprinic acid; Decoic acid; Decylic acid | Fatty Acyls [FA] | Fatty Acids and Conjugates [FA01] | Straight chain fatty acids [FA0101] | FA 10:0 |
| pos\_809 | Hexadecanedioic acid | LMFA01170022 | Thapsic acid | Hexadecanedioic acid | Thaspic acid | Fatty Acyls [FA] | Fatty Acids and Conjugates [FA01] | Dicarboxylic acids [FA0117] | FA 16:1;O2 |
| pos\_813 | Phytosphingosine | LMSP01030001 | Phytosphingosine | 4R-hydroxysphinganine | 4-hydroxysphinganine | Sphingolipids [SP] | Sphingoid bases [SP01] | 4-Hydroxysphinganines (Phytosphingosines) [SP0103] | SPB 18:0;O3 |
| pos\_818 | Avocadene 1-acetate | LMFA05000640 | Avocadene 1-acetate | 2,4-dihydroxyheptadec-16-en-1-yl acetate | - | Fatty Acyls [FA] | Fatty alcohols [FA05] | - | FOH 19:2;O3 |
| pos\_82 | 16-fluoro-7Z-hexadecenoic acid | LMFA01090049 | 16-fluoro-7Z-hexadecenoic acid | 16-fluoro-7Z-hexadecenoic acid | 16:1(7Z)(16F) | Fatty Acyls [FA] | Fatty Acids and Conjugates [FA01] | Halogenated fatty acids [FA0109] | - |
| pos\_825 | 1-(6Z,9Z,12Z-octadecatrienoyl)-glycero-3-phosphate | LMGP10050023 | PA(18:3(6Z,9Z,12Z)/0:0) | 1-(6Z,9Z,12Z-octadecatrienoyl)-glycero-3-phosphate | LPA(18:3) | Glycerophospholipids [GP] | Glycerophosphates [GP10] | Monoacylglycerophosphates [GP1005] | LPA 18:3 |
| pos\_83 | 8Z,11Z,14Z-heptadecatrienoic acid | LMFA01030756 | Norlinolenic acid | 8Z,11Z,14Z-heptadecatrienoic acid | C17:3n-3,6,9 | Fatty Acyls [FA] | Fatty Acids and Conjugates [FA01] | Unsaturated fatty acids [FA0103] | FA 17:3 |
| pos\_830 | Isoamyl 2-furonpropionate | LMFA07010913 | Isoamyl 2-furonpropionate | 3-methylbutyl 3-(furan-2-yl)propanoate | - | Fatty Acyls [FA] | Fatty esters [FA07] | Wax monoesters [FA0701] | WE 12:3;O |
| pos\_85 | Ipomeamaronol | LMFA05000668 | Ipomeamaronol | 1-[5-(furan-3-yl)-2-methyloxolan-2-yl]-5-hydroxy-4-methylpentan-2-one | - | Fatty Acyls [FA] | Fatty alcohols [FA05] | - | FOH 15:5;O3 |
| pos\_856 | N-Ethyl trans-2-cis-6-nonadienamide | LMFA08020193 | N-Ethyl trans-2-cis-6-nonadienamide | (2E,6Z)-N-ethylnona-2,6-dienamide | - | Fatty Acyls [FA] | Fatty amides [FA08] | N-acyl amines [FA0802] | NA 11:2 |
| pos\_86 | (11Z)-8,18-propano-retinal | LMPR01090045 | (11Z)-8,18-propano-retinal | - | - | Prenol Lipids [PR] | Isoprenoids [PR01] | Retinoids [PR0109] | - |
| pos\_87 | 9,12,15-Octadecatrien-1-ol | LMFA05000216 | 9,12,15-Octadecatrien-1-ol | 9,12,15-Octadecatrien-1-ol | - | Fatty Acyls [FA] | Fatty alcohols [FA05] | - | FOH 18:3 |
| pos\_871 | Chenodeoxycholic acid glycine conjugate | LMST05030008 | Glycochenodeoxycholic acid | N-(3alpha,7alpha-dihydroxy-5beta-cholan-24-oyl)-glycine | Chenodeoxycholic acid glycine conjugate; Chenoglycodeoxycholic acid | Sterol Lipids [ST] | Steroid conjugates [ST05] | Glycine conjugates [ST0503] | ST 24:1;O4;G |
| pos\_884 | Anandamide | LMFA08040056 | Anandamide-d4 (20:4, n-6) | N-(2-hydroxyethyl-1,1,2,2-d4)-5Z,8Z,11Z,14Z-eicosatetraenamide | Anandamide; Anandamide(20:4, n-6); N-arachidonoyl ethanolamine; arachidonoylethanolamide; Arachidonoyl-EA; Arachidonoyl ethanolamide; AEA | Fatty Acyls [FA] | Fatty amides [FA08] | N-acyl ethanolamines (endocannabinoids) [FA0804] | - |
| pos\_889 | PI(16:0/20:0) | LMGP06010942 | PI(16:0/20:0) | 1-hexadecanoyl-2-eicosanoyl-glycero-3-phospho-(1'-myo-inositol) | PI(36:0); PI(16:0\_20:0) | Glycerophospholipids [GP] | Glycerophosphoinositols [GP06] | Diacylglycerophosphoinositols [GP0601] | PI 36:0 |
| pos\_89 | Strobopinin | LMPK12140187 | Strobopinin | - | - | Polyketides [PK] | Flavonoids [PK12] | Flavanones [PK1214] | - |
| pos\_890 | 2'-O-Methylphaseollinisoflavan | LMPK12080015 | 2'-O-Methylphaseollinisoflavan | - | - | Polyketides [PK] | Flavonoids [PK12] | Isoflavans [PK1208] | - |
| pos\_892 | Amphotericin B | LMPK06000002 | Amphotericin B | - | - | Polyketides [PK] | Polyenes [PK06] | - | - |
| pos\_9 | Retinoic acid | LMPR01090019 | Retinoic Acid | 3,7-dimethyl-9-(2,6,6-trimethylcyclohexen-1-yl)nona-2E,4E,6E,8E-tetraenoic acid | Tretinoin/All-Trans Retinoic Acid | Prenol Lipids [PR] | Isoprenoids [PR01] | Retinoids [PR0109] | - |
| pos\_90 | Peltogynan | LMPK12020154 | Peltogynan | - | - | Polyketides [PK] | Flavonoids [PK12] | Flavans, Flavanols and Leucoanthocyanidins [PK1202] | - |
| pos\_914 | TG(18:3(9Z,12Z,15Z)/20:4(5Z,8Z,11Z,14Z)/22:6(4Z,7Z,10Z,13Z,16Z,19Z))[iso6] | LMGL03012094 | TG(18:3(9Z,12Z,15Z)/20:4(5Z,8Z,11Z,14Z)/22:6(4Z,7Z,10Z,13Z,16Z,19Z))[iso6] | 1-(9Z,12Z,15Z-octadecatrienoyl)-2-(5Z,8Z,11Z,14Z-eicosatetraenoyl)-3-(4Z,7Z,10Z,13Z,16Z,19Z-docosahexaenoyl)-sn-glycerol | TG(18:3/20:4/22:6)[iso6]; TG(60:13); TG(18:3\_20:4\_22:6) | Glycerolipids [GL] | Triradylglycerols [GL03] | Triacylglycerols [GL0301] | TG 60:13 |
| pos\_919 | 4-Hydroxyandrostenedione glucuronide | LMST05010040 | 4-Hydroxyandrostenedione glucuronide | - | - | Sterol Lipids [ST] | Steroid conjugates [ST05] | Glucuronides [ST0501] | ST 19:3;O3;GlcA |
| pos\_932 | Falcarindiol | LMFA05000658 | Falcarindiol | (3R,8S,9Z)-heptadeca-1,9-dien-4,6-diyne-3,8-diol | (3R,8S)-Falcarindiol; (3R,8S,9Z)-heptadeca-1,9-dien-4,6-diyne-3,8-diol; (3S,8S)-Falcarindiol; AC1NQY3Z | Fatty Acyls [FA] | Fatty alcohols [FA05] | - | FOH 17:6;O |
| pos\_961 | 2-Octenedioic acid | LMFA01170085 | 2-octenedioic acid | 2E-octenedioic acid | - | Fatty Acyls [FA] | Fatty Acids and Conjugates [FA01] | Dicarboxylic acids [FA0117] | FA 8:2;O2 |
| pos\_97 | 5-(1-oxopropan-2-yl)isolongifol-5-ene | LMPR0103500011 | 5-(1-oxopropan-2-yl)isolongifol-5-ene | 2-[(1S,8S)-2,2,7,7-tetramethyltricyclo[6.2.1.0(1,6)]undec-5-en-5-yl]propanal | 2-(2,2,7,7-tetramethyltricyclo[6.2.1.0(1,6)]undec-5-en-5-yl)propanal | Prenol Lipids [PR] | Isoprenoids [PR01] | C15 isoprenoids (sesquiterpenes) [PR0103] | - |
